# Supplementary material for: Effects of continuous positive airway pressure on neurocognitive architecture and function in patients with obstructive sleep apnoea: study protocol for a multicentre randomised controlled trial
Source: BMJ Open. 2017 May 25;7(5):e014932. doi: 10.1136/bmjopen-2016-014932 (PMC5729992; doi:10.1136/bmjopen-2016-014932)
Supplement: Author's manuscript [file bmjopen-2016-014932.draft_revisions.pdf]

BMJ Open

# Effects of Continuous Positive Airway Pressure on Cognitive Function, Neurocognitive Architecture and Function in Patients with Obstructive Sleep Apnea: The Shanghai Multicenter Obstructive Sleep Apnea Therapy Trial


| | |
|---|---|
| Journal: | *BMJ Open* |
| Manuscript ID | bmjopen-2016-014932 |
| Article Type: | Protocol |
| Date Submitted by the Author: | 01-Nov-2016 |



| | |
|---|---|
| Complete List of Authors: | Xu, Huajun; Shanghai 6th Peoples Hospital Affiliated to Shanghai Jiaotong University School of Medicine, Department of Otolaryngology Head and Neck Surgery & Center of Sleep Medicine<br>Wang, Hui; Shanghai 6th Peoples Hospital Affiliated to Shanghai Jiaotong University School of Medicine, Department of Otolaryngology Head and Neck Surgery & Center of Sleep Medicine<br>Guan, Jian; Shanghai 6th Peoples Hospital Affiliated to Shanghai Jiaotong University School of Medicine, Department of Otolaryngology Head and Neck Surgery & Center of Sleep Medicine<br>Yi, Hongliang; Shanghai 6th Peoples Hospital Affiliated to Shanghai Jiaotong University School of Medicine, Department of Otolaryngology Head and Neck Surgery & Center of Sleep Medicine<br>Qian, Yingjun; Shanghai 6th Peoples Hospital Affiliated to Shanghai Jiaotong University School of Medicine, Department of Otolaryngology Head and Neck Surgery & Center of Sleep Medicine<br>Zou, Jianyin; Shanghai 6th Peoples Hospital Affiliated to Shanghai Jiaotong University School of Medicine, Department of Otolaryngology Head and Neck Surgery & Center of Sleep Medicine<br>Xia, Yunyan; Shanghai 6th Peoples Hospital Affiliated to Shanghai Jiaotong University School of Medicine, Department of Otolaryngology Head and Neck Surgery & Center of Sleep Medicine<br>Fu, Yiqun; Shanghai 6th Peoples Hospital Affiliated to Shanghai Jiaotong University School of Medicine, Department of Otolaryngology Head and Neck Surgery & Center of Sleep Medicine<br>Li, Xinyi; Shanghai 6th Peoples Hospital Affiliated to Shanghai Jiaotong University School of Medicine, Department of Otolaryngology Head and Neck Surgery & Center of Sleep Medicine<br>Jiao, Xiao; Shanghai 6th Peoples Hospital Affiliated to Shanghai Jiaotong University School of Medicine, Department of Otolaryngology Head and Neck Surgery & Center of Sleep Medicine<br>Huang, Hengye; Shanghai Jiao Tong University, Department of Epidemiology, School of Public Health<br>Dong, Pin; Shanghai Jiaotong University First People's Hospital, Department of Otorhinolaryngology<br>YU, Ziwei; Shanghai Jiaotong University First People's Hospital, Department of Otorhinolaryngology<br>Yang, Jun; Shanghai Jiaotong University School of Medicine Xinhua Hospital, Department of Otolaryngology Head and Neck Surgery<br>Xiang, Mingliang; Shanghai Jiaotong University School of Medicine Xinhua |

| | |
|---|---|
| | Hospital, Department of Otolaryngology Head and Neck Surgery<br>Li, Jiping; Shanghai Jiao Tong University School of Medicine Affiliated Renji Hospital, Department of Otolaryngology Head and Neck Surgery<br>Chen, Yanqing; Shanghai Jiao Tong University School of Medicine Affiliated Renji Hospital, Department of Otolaryngology Head and Neck Surgery<br>Wang, Peihua; Shanghai 9th Peoples Hospital Affiliated to Shanghai Jiaotong University School of Medicine, Department of Otolaryngology Head and Neck Surgery<br>Sun, Yizhou; Shanghai 9th Peoples Hospital Affiliated to Shanghai Jiaotong University School of Medicine, Department of Otolaryngology Head and Neck Surgery<br>Li, Yuehua; Shanghai 6th Peoples Hospital Affiliated to Shanghai Jiaotong University School of Medicine, Department of Radiology<br>Zheng, Xiaojian; Shanghai 6th Peoples Hospital Affiliated to Shanghai Jiaotong University School of Medicine, Shanghai Key Laboratory of Diabetes Mellitus and Center for Translational Medicine<br>Jia, Wei; Shanghai 6th Peoples Hospital Affiliated to Shanghai Jiaotong University School of Medicine, Shanghai Key Laboratory of Diabetes Mellitus and Center for Translational Medicine<br>Yin, Shankai; Shanghai 6th Peoples Hospital Affiliated to Shanghai Jiaotong University School of Medicine, Department of Otolaryngology Head and Neck Surgery & Center of Sleep Medicine |
| <b>Primary Subject Heading</b>: | Ear, nose and throat/otolaryngology |
| Secondary Subject Heading: | Ear, nose and throat/otolaryngology, Neurology |
| Keywords: | obstructive sleep apnea, continuous positive airway pressure, cognitive function, gut microbiota, metabolomics |
| | |

SCHOLARONE™
Manuscripts

view only

# Effects of Continuous Positive Airway Pressure on Cognitive Function, Neurocognitive Architecture and Function in Patients with Obstructive Sleep Apnea: The Shanghai Multicenter Obstructive Sleep Apnea Therapy Trial

(Running title: Effects of CPAP on Cognitive Function, Neurocognitive Architecture and Function in Patients with OSA: The SMOSAT Trial)

Xu H MD,Ph D[1,2,3*], Wang H MD,Ph D [1,2,3*], Guan J MD,Ph D [1,2,3], Yi H MD, Ph D [1,2,3], Qian Y MD,Ph D [1,2,3], Zou J MD,Ph D [1,2,3], Xia Y MD [1,2,3], Fu Y MD[1,2,3], Li X MD[1,2,3], Jiao X MD[1,2,3], Huang H MD [4], Dong P MD,Ph D [5], Yu Z, MD,Ph D [5], Yang J MD,Ph D[6], Xiang M MD,Ph D[6], Li J MD,Ph D [7], Chen Y MD,Ph D [7],Wang P MD,Ph D [8], Sun Y, MD,Ph D [8], Li Y MD,Ph D[9], Zheng X,Ph D [10], Jia W MD,Ph D [10,11] , Yin S MD,Ph D [1,2,3]

Both Xu H and Wang H contributed equally to this paper

Corresponding to Yin S, skyin@sjtu.edu.cn

1 Department of Otolaryngology Head and Neck Surgery & Center of Sleep Medicine, Shanghai Jiao Tong University Affiliated Sixth People's Hospital, 600 Yishan Road, 200233 Shanghai, China.

2 Otolaryngological Institute of Shanghai Jiao Tong University, 600 Yishan Road, 200233 Shanghai, China.

3 Clinical Research Center, Shanghai Jiao Tong University School of Medicine, 225 South Chongqing Road, 200020 Shanghai, China.

4 Department of Epidemiology, School of Public Health, Shanghai Jiao Tong University, 225South Chongqing Road, 200020 Shanghai, China.

5Department of Otorhinolaryngology, Shanghai First People's Hospital, Shanghai Jiao Tong

For peer review only

University, 100 Haining Road, 200080 Shanghai, China.

6 Department of Otolaryngology Head and Neck Surgery, Xinhua Hospital, Affiliated to Shanghai Jiaotong University School of Medicine, 1665 Kongjiang Road, 200092 Shanghai, China.

7 Department of Otorhinolaryngology Head and Neck Surgery, Ren Ji Hospital, School of Medicine, Shanghai Jiao Tong University, 160 Pu Jian Road, Shanghai, 200127, China.

8 Department of Otolaryngology Head and Neck Surgery, Shanghai Ninth People's Hospital, Affiliated to Shanghai Jiaotong University School of Medicine, 500 Quxi Road, Shanghai, 200000, China.

9 Department of Radiology, Shanghai Jiao Tong University Affiliated Sixth People's Hospital, 600 Yi Shan Road, Shanghai 200233, China.

10 Shanghai Key Laboratory of Diabetes Mellitus and Center for Translational Medicine, Shanghai Jiao Tong University Affiliated Sixth People's Hospital, , 600 Yishan Road, Shanghai, 200233, China.

11 University of Hawaii Cancer Center, Honolulu, Hawaii, 96813, USA.

For peer review only

## Abstract

### Objectives

Multiple clinical studies have indicated that obstructive sleep apnea (OSA), the most common chronic sleep disorder, may affect neurocognitive function, and that treatment for continuous positive airway pressure (CPAP) has some neurocognitive protective effects against the adverse effects of OSA. However, the effects of CPAP treatment on neurocognitive architecture and function remain unclear. Therefore, this multicenter trial was designed to investigate whether and when neurocognitive architecture and function in patients with OSA can be improved by CPAP treatment, and to explore the role of gut microbiota in improving neurocognitive function during treatment.

### Methods/Design

This study will be a multicenter, randomized, controlled trial with allocation concealment and assessor blinding. A total of 148 eligible patients with severe OSA will be enrolled from five sleep centers, and randomized to receive CPAP with best supportive care (BSC) intervention or BSC intervention alone. Cognitive function, structure and function of brain regions, gut microbiota, metabolites, biochemical variables, electrocardiography, echocardiography, pulmonary function, and arterial stiffness will be assessed at baseline before randomization and at 3, 6, and 12 months. In addition, we will enroll 74 healthy controls and assess all of the aforementioned variables at baseline.

### Ethics and Dissemination

Ethics approval was given by the Medical Ethics Committee of Shanghai Jiao Tong University Affiliated Sixth People’s Hospital (approval number 2015-79). The findings from this study will be disseminated in peer-reviewed journals and at conferences.


For peer review only

**Trial registration number:** NCT02886156; Pre-results.

**Keywords:** obstructive sleep apnea, continuous positive airway pressure, cognitive function, gut microbiota, metabolomics

**Article summary**

Strengths and limitations of this study:

1) Our study will evaluate the effects of CPAP on neurocognitive architecture and function dynamically.

2) This study will also evaluate metabolic status and gut microbiota dynamically after CPAP treatment.

3) This trial will be designed to explore the role of gut microbiota in improving neurocognitive function and metabolic disorders during treatment.

4) A prospective, multicenter randomized, controlled study with blinded outcome measurements will be performed. A broad measurement tool and multiple measurement time points will be used to judge the efficiency of CPAP treatment.

5) The efficacy of long-term CPAP intervention in patients with OSA in improving neurocognitive architecture and function remains to be determined.

For peer review only

**Background**

Obstructive sleep apnea (OSA) is a common chronic sleep disorder characterized by recurrent partial or complete pharyngeal obstruction during sleep [1]. There is a high prevalence (2–4%) of this condition in adults aged 30–60 years, which is increasing with the growing epidemic of obesity [2-4]. OSA is well recognized as a risk factor for neurocognitive, cardiovascular, and metabolic disorders [5-7]. It is worth noting that neurocognitive dysfunction is associated with a series of sequelae, such as social and economic burdens [8-10]. Continuous positive airway pressure (CPAP) is typically recommended as first-line treatment for OSA, as it can partly or completely reverse OSA-related complications, including cognitive impairments [11 12]. In addition, CPAP may be effective for reversing structural and functional brain changes [13 14].

To date, there have only been two randomized controlled trials (RCTs) that simultaneously evaluated the effects of CPAP on cognition and neuroimaging; both studies had relatively small sample sizes and short intervention periods [15 16]. However, the response to CPAP treatment in the trials was variable. Thus, the relationships among OSA, brain, and neurobehavioral function are complex and must be comprehensively evaluated, including how, when, and how long CPAP treatment should be applied for effective therapy.

The gut microbiota is an important factor that affects neurological functions [17], and may be involved in the modulation of central nervous function (e.g., the hypothalamus–pituitary–adrenal [HPA] axis) and associated with neuropsychiatric conditions [17 18]. The composition of the gut microbiota is altered through intermittent hypoxia or sleep fragmentation in animal models [19 20]. However, there have been no studies to determine whether changes in human intestinal microbiota are associated with OSA, and whether or how the microbiota can be modulated by CPAP. This study will clarify the complex

For peer review only

relationships among OSA, gut microbiota, and neurological functions.

## Methods/Design

### *Study aims and hypotheses*

The primary aim of this study is to assess the dynamic (3, 6, and 12 months) effects of CPAP on cognitive function, neurocognitive architecture and function in patients with OSA. The primary hypothesis of this study is that patients with OSA will show improved cognitive function through structural and functional brain modulation immediately after 3 months of CPAP treatment, and that these benefits will continue at the 6-month and 12-month follow-ups. The secondary aims of this study are to investigate whether and which sleep parameters are correlated with improvements of cognitive function, neurocognitive architecture and function; to explore whether the metabolic status assessed by metabolomics can be reversed by CPAP and correlated with improvement of neurocognitive impairment; and to investigate whether CPAP modulates the gut microbiota and contributes to alleviation of neurocognitive impairment. We hypothesize that patients with OSA who undergo CPAP treatment will have metabolic improvement and modulation of gut microbiota, and that these changes will have beneficial effects on neurocognitive impairment.

*Study design*

The Shanghai Multicenter Obstructive Sleep Apnea Therapy trial is a multicenter, evaluator-blinded, randomized, parallel controlled trial performed in five sleep centers (Department of Otolaryngology & Head and Neck Surgery, Shanghai Jiao Tong University Affiliated Sixth People's Hospital; Department of Otolaryngology-Head and Neck Surgery, Shanghai First People's Hospital, Shanghai Jiao Tong University; Department of

For peer review only

Otolaryngology, Renji Hospital, School of Medicine, Shanghai Jiaotong University; Department of Otolaryngology, Head and Neck Surgery, Xinhua Hospital, Shanghai Jiaotong University School of Medicine; Department of Otolaryngology, The Ninth People's Hospital Affiliated to Shanghai Jiao-Tong University School of Medicine) in the Shanghai district. The design of this protocol strictly followed the Standard Protocol Items Recommendations for Interventional Trials [21] and the Consolidated Standards of Reporting Trials statements [22]. A total of 148 eligible participants with severe OSA will be enrolled from the abovementioned five sleep centers and randomized to receive CPAP with best supportive care (BSC) intervention or BSC intervention alone. Cognitive function, structure and function of brain regions, metabolites, gut microbiota, biochemical variables, electrocardiography, echocardiography, pulmonary function, and arterial stiffness will be assessed at baseline before randomization and at 3, 6, and 12 months. In addition, 74 age-, body mass index (BMI)-, and education year-matched participants (healthy controls) will be enrolled and all of the aforementioned variables will be assessed at baseline. The flow diagram of this RCT is presented in Figure 1.

**Study population**

The study population (30–65 years old) will be enrolled from Shanghai and the surrounding area, and all of the participants will be from the Han population. The diagnostic, inclusion, and exclusion criteria for this trial will be as described below.

*Diagnostic criteria*

According to the American Academic Sleep Medicine (AASM) criteria, severe OSA is defined as an apnea-hypopnea index (AHI) $\geq$ 30 events per hour, and the normal state is

For peer review only

defined as AHI < 5 events per hour.

*Eligible participants*

*Inclusion criteria*

1) Informed consent
2) Age 30–65 years
3) Newly diagnosed OSA (full-night in-laboratory polysomnography [PSG] with AHI ≥ 30 events per hour)
4) Adherence to CPAP treatment
5) No participation in any other clinical trial in the past 3 months
6) Able to accomplish relevant tests and follow-up

*Exclusion criteria*

1) Severe systemic diseases (e.g., cardiac, hepatic, and renal failure)
2) Psychiatric conditions (e.g., depression, mania, schizophrenia)
3) Neurological diseases (e.g., head trauma, brain tumor, epilepsy, stroke, transient ischemic attack, coma)
4) Sleep disorders other than OSA (narcolepsy, insomnia, chronic sleep deprivation, rapid eye movement [REM] behavior disorder and restless legs syndrome, central sleep apnea or obesity hypoventilation syndrome)
5) Alcoholism, drug addiction, use of psychotropic drugs, sedatives, or narcotics
6) Prior therapy for OSA (i.e., CPAP, upper airway surgery, oral appliance)
7) Minimum Mental State Examination (MMSE)< 24
8) Left-handed
9) MRI contraindications (e.g., claustrophobic or metal implantation)

For peer review only

10) Gastrointestinal surgery during the last year, except for appendicitis and hernia surgery

11) Pregnancy

12) Use of intestinal flora regulator (e.g., antibiotics or probiotics) in the previous 8 weeks

13) Medical treatment for cholecystitis, gallstones, gastrointestinal ulcers, urinary tract infection, acute pyelonephritis, or cystitis in the past 3 months

14) Infectious diseases, such as tuberculosis, acquired immune deficiency syndrome

15) Deemed by the researchers to be suitable for this trial

**Withdrawal criteria and management**

Subjects will be allowed or required to withdraw from this trial for adverse events related to CPAP, major protocol violation, or the development of serious disease that prevents continuation in the trial.

**Recruitment and screening**

Participants will be recruited from Shanghai and its vicinity through newspapers and instant messaging software (WeChat). Potential participants with self-reported severe snoring and with an interest in participating will be screened by a standard PSG and examined for eligibility (AHI ≥ 30 times per hour) for multicenter RCT. Potential participants with no sleep complaints will also be screened by a standard PSG and examined for eligibility (AHI < 5 times per hour) as controls. Eligible participants will be given information about this trial and have an informed discussion with trained research assistants regarding the information provided. Written informed consent will be obtained from each participant prior to enrollment.

For peer review only

**Randomization, allocation concealment, and blinding**

After baseline assessment, eligible patients with severe OSA will be randomly and equally allocated to the CPAP plus BSC group or BSC only group. The central random allocation sequence will be produced using the statistical software SAS v.9.4 and managed by an independent statistician blinded to the recruitment, evaluation, and intervention of the participants. The research assistant from each sleep center will request (via E-mail) a participant's allocation from the statistician. The group allocation will not be masked by participants or intervention supervisors in this trial; however, it can be blinded to outcome assessors and data statisticians.

**Intervention**

*BSC only group*

Participants in the BSC only group will receive advice regarding lifestyle modification, sleep hygiene, naps, exercise, caffeine, and diet, and avoiding alcohol consumption, but no specific weight loss program, diet, or salt restriction will be suggested.

*CPAP plus BSC group*

Participants in the CPAP plus BSC group will receive CPAP treatment plus the aforementioned BSC intervention. CPAP treatment (LOTUS AUTO; Curative Medical Technology Inc., Beijing, China) will be initiated using standard clinical practice at each center. Participants in both groups will be asked to continue their usual medical care during the trial.

**Follow-up**

At 3, 6, and 12 months during the intervention period, all of the participants will undergo the

For peer review only

same examinations as at baseline. During the follow-up period, CPAP usage information will be automatically downloaded daily from auto CPAP via the Internet.

**Outcome assessment**

The variables in this trial consist of basic characteristics, primary outcomes, and secondary outcomes. The basic characteristics will be measured at baseline, and primary and secondary outcomes will be measured at baseline and at the end of each intervention period (3, 6, and 12 months after randomization). All of the primary and secondary outcomes will be assessed by experienced staff at each sleep center who will be blinded to the allocation results of participants.

**Basic characteristics**

Participants' demographic characteristics (e.g., age, sex, years of education, medical history, medication use, occupation) will be collected by recruiters using a questionnaire. The basic Montreal Cognitive Assessment (MoCA) and Minimum Mental State Examination (MMSE) scores will be assessed using the corresponding standardized scales. The assessors in each sleep center will be trained by a neurologist. Baseline assessment will be completed before randomization.

**Primary and secondary outcomes**

*Primary endpoints*

Neurocognitive function (MoCA and MMSE), structure and function of related brain regions) will be measured using fMRI.

*Secondary endpoints*

Neuropsychological tests includes mental arithmetic, memory scanning, movement

For peer review only

perception, switching attention, space location memory span, attention allocation choice reaction time curve fit will be also performed.

Sleep variables (apnea-hypopnea index [AHI], oxygen desaturation index [ODI], lowest oxygen saturation [LSa$O_2$)] mean oxygen saturation [mean Sa$O_2$], micro-arousal index [MAI])

Auditory Testing

Central auditory processing testing (Speech-in-noise test, Competing sentences test, Gap detection, Dichotic listening)

256-channel high-density EEG recordings

Intestinal bacterial diversity and community structure will be detected by Metagenomics approch

Metabolomics profiling: metabolites will be detected by a metabolomics approach

Amyloid β protein 42, pre-senilin 1, amyloid-β protein 40, total GSK-3β, and serine-9 phosphorylated GSK-3β

*Other outcomes*

Biochemical variables: lipid profile (total cholesterol, triglycerides, high-density lipoprotein cholesterol, low-density lipoprotein cholesterol, apoA-I, apoB, apoE, and lipoprotein(a)), glucose, insulin, HbA1c, free fatty acids, cholinesterase, lipopolysaccharide binding protein, toll-like receptor-4, TLR-2, hepatic function tests (alanine aminotransferase, aspartate aminotransferase, γ-glutamyl endopeptidase, alkaline phosphatase, total protein, albumin, total bilirubin, direct bilirubin, bile acid, prealbumin), renal function tests (creatinine, urea nitrogen, uric acid), microalbuminuria; apolipoprotein E genotyping

Inflammatory biomarkers: leptin, RANTES, monocyte chemotactic factor 1, interleukin

For peer review only

(IL)-6, IL-8, tumor necrosis factor-α, hypersensitive C-reactive protein, resistin, serum amyloid A protein, α-acid glycoprotein

Anti-inflammatory biomarkers: adiponectin, IL-1Ra

Oxidative stress index: 8-isoprostane

Blood pressure (systolic blood pressure, diastolic blood pressure)

Anthropometric variables (weight, height, waist, hip, chest and neck circumferences, visceral fat area, subcutaneous fat area)

Echocardiography

Arterial stiffness (pulse wave velocity, ankle brachial index, toe-brachial index)

Lung function (e.g., forced expiratory volume in 1 s, forced vital capacity)

Electrocardiography (ECG)

**Study examination procedures**

All of the study measurements will be performed before and after 3, 6, and 12 months of CPAP intervention at each center except for lung function and ECG.

*Anthropometric measurements*: Height and weight will be measured using standard methods. BMI (weight [kg]/height$^2$[m$^2$]) will be calculated from height and weight. Waist, hip, and neck circumferences will be measured using a measuring tape in the conventional manner.

*Urine samples*: First midstream urine samples will be collected in the morning with the collected urine samples stored immediately at –80°C for further analyses.

*Blood samples*: Fasting blood samples will be collected in the morning, and the samples will be immediately sent for further analyses such as biochemical analysis.

*Fecal samples*: Fecal samples will be collected and stored at –80°C until use in subsequent assays. The composition of microbiota will be measured using 16S rRNA-hybridization and

For peer review only

DNA staining.

*Sleep respiratory variables*: Laboratory-based PSG (Alice 4 or 5, Respironics; Pittsburgh, PA, USA) will be used to record sleep parameters. Electroencephalography (EEG), electrooculography, ECG, electromyography, nasal/oral airflow, thoracic/abdominal movement, pulse oximetry, body posture, and snoring will be recorded. The definition of OSA is established according to the AASM criteria.

*Neuropsychological tests*: Two questionnaires (MoCA and MMSE) will be administered. The neuropsychological tests includes mental arithmetic, memory scanning, movement perception, switching attention, space location memory span, attention allocation choice reaction time curve fit will be also performed.

*Auditory testing*: Audiograms will be measured in one-octave steps at frequencies ranging from 125 Hz to 8 kHz and then at 10, 12.5, 16, and 20 kHz. DPOAE values will be recorded in both groups using a Madsen Capella cochlear emissions analyzer in a soundproof room. Only patients with normal audiometry (threshold below 25 dB HL at all frequencies from 0.125 to 20 kHz), normal DPOAE levels, normal tympanometric curve of type A, and presence of ipsi- and contralateral stapedial reflexes will be included.

*Central auditory processing testing*: The following central auditory processing tests will be performed in a soundproof room. Before the formal test, participants will be allowed to practice as many times as required and provide feedback to become familiar with the stimuli. (1) Speech-in-noise test: All of the signal processing will be performed with MATLAB software. The speech of 20 ten-word sentences will be presented under both quiet and noisy conditions with speech-shaped noise, and will be delivered bilaterally through Sennheiser HD205 headphones at a signal-to-noise ratio of –5 dB. (2) Competing sentences test: Babble

For peer review only

noise and the target sentence will be presented bilaterally at an intensity level of 65 dB SL (above speech reception threshold) at a signal-to-noise ratio of –3 dB. (3) Gap detection: The purpose of this evaluation is to determine the smallest interval that the listener can detect. Two stimuli separated in time by a "gap" will be presented and the listener will be asked to detect the gap, essentially discriminating sound from silence. Stimuli as white noise will be bilaterally presented as background noise at intensities 40 dB higher than the hearing threshold. (4) Dichotic listening: Two different numbers will be simultaneously presented to both ears with the subjects instructed to repeat both numbers in any order. The test includes 20 number pairs presented dichotically. The number pairs will be aligned for onset and offset to eliminate any cue for the first number heard. Dichotic listening performance will be scored as the percentage of correctly reported different numbers from the right and left ears in the noise-exposed and control groups.

*256-Channel high-density EEG recording*: EEG data will be recorded with 256 channels on EGI's HydroCel Geodesic Sensor Net, with Cz used as the reference channel. All of the electrode impedances will be detected and maintained at less than 50 kΩ. The oddball stimuli pattern with speech sound is used in this study (via Eprime 2.0; Psychology Software Tools). The auditory oddball task includes presentation sound of /Ba/, whereas /Da/ is presented as the "oddball" target stimulus. The stimulus /Ba/ is presented in 85% of the trials together with the stimulus /Da/ in 15% of the trials. The whole task consists of a total of 1000 auditory stimuli with random interstimulus intervals ranging from 850 ms to 1450 ms. The sound stimuli with an intensity of 75 dB delivered through two loudspeakers at a distance of 100 cm from the subjects. During the P3 cortical waves test, the subjects will be asked to press a switch held in the right hand each time they detect a target tone with the eyes closed. In

For peer review only

addition, they will be asked not to respond to any auditory stimulus while watching silent films during the MMN test. Source analysis of event-related potentials as MMN, describing neural sources of measured scalp potentials, will be estimated with GeoSource (version 2.0).

*Brain imaging:* All the MRI scans were performed on a Siemens 3T Verio scanner (MAGETOM, Siemens) using a 12-channel coil using the same parameters. A high-resolution T1-3D, T2-3D weighted sequences MRI scan was conducted for all the subjects. T1 3D axial images: FOV, 230 mm; TR/TE, 1500/2.96 ms; flip angle, 9°; voxel size, $0.9\times0.9\times1mm^3$; slice thickness, 1 mm; and distance factor, 50%. T2 3D axial images: FOV, 230 mm; TR/TE,1500/2.96 ms; voxel size, $0.9\times0.9\times1mm^3$; slice thickness, 1 mm; and distance factor, 50%. At the same position, rs-fMRI BOLD sequence acquired during the rest condition. 240 functional images were obtained (repetition time 2,000 m sec, echo time 30 m sec, thickness 4.0 mm, gap 1.2 mm, acquisition matrix 64×64, flip angle 90 degrees, field of view 230 mm ×230 mm, 30 axial slices with gradient-recalled echo-planar imaging pulse sequence covering the whole brain). Patients were asked to keep their eyes closed and not to focus their thoughts on any particular topic and not to fall asleep. DKI was performed in all subjects using 30 gradient directions, six b values and an echo-planar DWI sequence. FOV, 300 mm; TR/TE, 1100/109 ms; matrix size 128×128; and slice thickness: 4 mm.

*Cerebral metabolites*: MRS scans were performed on a Siemens 3T Verio scanner using a 12-channel coil. A $2.0\times2.0\times2.0\ cm^3$ voxel of interest (VOI) was placed at the frontal lobe . NAA, Cho, and Cr were acquired by a MEGA-PRESS sequence with the following parameters: TE/TR=135/5000 ms; number of average =80 (scan time= 8 min).

*Abdominal and liver fat measurements:* The hepatic, subcutaneous fat and visceral fat were measured through axial scanning in upper abdomen (from diaphragm to the lower edge of

For peer review only

liver). The participants should hold to breath in order to reduce the artifacts induced by respiratory movement. Anatomical T1-weighted spin-echo MR images were obtained using the following parameters: slice thickness = 5 mm; slice spacing = 2.5 mm.

**Safety measurements**

Any unexpected adverse events during the intervention period will be monitored, reported to a research assistant, and causality in relation to CPAP intervention and the severity of adverse events will be analyzed. Serious adverse events will be immediately reported to the ethics committee.

**Sample size calculation**

A previous study indicated that the mean and standard deviation (SD) of MoCA score are 26.1 (2.0) and 27.4 (2.3) before and after CPAP treatment, respectively [23]. With a significance level of 0.05 and statistical power of 0.9, the calculated sample size is 59 for each group. Considering a 20% loss to follow-up, a total of 148 participants will be required for randomization.

**Statistical methods**

All of the statistical analyses will be performed using SAS v.9.4 software, with a two-sided p value $< 0.05$ indicating statistical significance. The Kolmogorov–Smirnov test will be performed to test normality of the distribution. Continuous variables will be expressed as means and SD or median (first third quartile) for continuous variables and percentages for categorical variables. Primary and secondary outcomes will be analyzed by intention-to-treat (ITT) and per-protocol. A multiple imputation method will be used to fill in missing data.

For peer review only

## Data collection and management

All of the data collectors and evaluators will be blinded to the group assignments. The data in this trial will be collected using print-based case report forms, which will be transcribed by a research assistant into web-based case report forms (w-CRFs) with an electronic data capture system (EDC). The EDC will be provided by the School of Public Health of Shanghai Jiao Tong University (Shanghai, China) and will meet the available standards of security. Data in w-CRFs will be stored in the EDC system at separate password-protected locations. The research assistants will be responsible for ensuring the integrity and accuracy of all data when data are transcribed into w-CRFs by checks on value ranges and logical checks. Development and validation of the study database, quality control, and extraction of data will be performed according to the procedures of the medical research platform.

## Ethical issues

This protocol will be conducted in accordance with the Declaration of Helsinki. Ethics approval has been obtained from Shanghai Jiao Tong University Affiliated Sixth People's Hospital (Ethics number: 2015–79). Written informed consent will be obtained from each participant with a full discussion about the potential benefits and risks before participation.

## Dissemination

This study will be published in scientific journals and presented at national conferences in the field of neurology and sleep medicine. Study results will also be sent to study participants and disseminated to researchers, healthcare providers, healthcare professionals, and the general public regardless of the magnitude or direction of effects.

For peer review only

**Discussion**

Increasing numbers of clinical studies have shown that CPAP is an effective therapeutic method for neurocognitive dysfunction in OSA. However, the effects and duration of CPAP treatment on neurocognitive function have not been clearly established. This study focuses on the effects of CPAP on middle-aged patients with severe OSA. Through a 3-, 6-, and 12-month intervention with CPAP treatment, the results from a range of clinical and biochemical assessments will provide clearer information on the effects of treatment in OSA.

Measurements of neurocognitive function before and after intervention in this trial may strengthen evidence for links between OSA and neurocognitive dysfunction, or reveal new associations. Comparing patients with OSA to healthy controls will allow us to identify differences in neurocognitive function between the two groups. In addition, through CPAP intervention, it will be possible to explore the mechanism of action of CPAP on neurocognitive function (including structural and functional effects), allowing us to investigate the causal relationships between OSA and neurocognitive dysfunction. Through metagenomics and metabolomics approaches, we will determine whether gut microbiota play a pivotal role in OSA and neurocognitive dysfunction.

To the best of our knowledge, the proposed study will be the first to investigate the effects of CPAP on neurocognitive dysfunction in OSA and to explore the underlying mechanisms using combined "-omics" approaches. The results of this multicenter trial may be helpful for improving the neurocognitive function of patients with OSA.

This protocol describes a multicenter RCT to assess the effects of CPAP treatment on neurocognitive function in patients with severe OSA. To reduce potential bias, multiple rigorous methods, such as randomization, blinding of raters and statistical analyzers, and ITT

For peer review only

principles, will be implemented. Potential limitations of this protocol should also be addressed. First, sham CPAP is not used as a placebo; therefore neither participants nor trial researchers will be blinded to the protocol. Thus, performance bias may be inevitable. Second, although all of the raters have been trained by the same neurologist, there may be heterogeneity in neuropsychological tests in each center.

In summary, this will be the first multicenter RCT to dynamically observe the effects of CPAP on neurocognitive function and to explore the role of the gut microbiota in neurocognitive dysfunction in OSA. The results of this study will help to clarify potential mechanisms for alleviation of OSA and improvement of neurocognitive dysfunction.

**Trial status:** At the time of manuscript submission, the enrollment of volunteers is ongoing.

**Acknowledgements** This study was supported by grants-in-aid from multi-center clinical research project from school of medicine, Shanghai Jiao Tong University (DLY201502). The study sponsor played no role in the design, methods, data management or analysis or in the decision to publish. The corresponding authors have full access to all the data in the study and have final responsibility for the decision to submit for publication.

**Contributors** Xu H, Wang H and Yin S conceived the study, designed the study protocol, and drafted the manuscript. Xu H, Wang H, Guan J, Yi H, Qian Y, Zou J, Xia Y, Fu Y, Li X, Jiao X, Dong P, Yu Z, Yang J, Xiang M, Li J, Chen Y, Wang P, Sun Y, Li Y, Jia W and Yin S participated in the coordination and implementation of the study. Xu H, Wang H and Yin S revised and finalized the study protocol. Yin S is in charge of coordination and direct implementation. Huang H helped to develop the study measures and analyses. All authors contributed to drafting the manuscript and have read and approved the final manuscript.

**Competing interests** The authors declare that they have no competing interests.

For peer review only

**Patient consent** Obtained.

**Ethics approval** Obtained.

**References:**


1. Strollo PJ, Jr., Rogers RM. Obstructive sleep apnea. The New England journal of medicine 1996;**334**(2):99-104.
2. Young T, Palta M, Dempsey J, et al. The occurrence of sleep-disordered breathing among middle-aged adults. The New England journal of medicine 1993;**328**(17):1230-5.
3. Peppard PE, Young T, Barnet JH, et al. Increased prevalence of sleep-disordered breathing in adults. Am J Epidemiol 2013;**177**(9):1006-14.
4. Heinzer R, Vat S, Marques-Vidal P, et al. Prevalence of sleep-disordered breathing in the general population: the HypnoLaus study. The Lancet Respiratory medicine 2015;**3**(4):310-8.
5. Sanchez-de-la-Torre M, Campos-Rodriguez F, Barbe F. Obstructive sleep apnoea and cardiovascular disease. The Lancet Respiratory medicine 2013;**1**(1):61-72.
6. Aurora RN, Punjabi NM. Obstructive sleep apnoea and type 2 diabetes mellitus: a bidirectional association. The Lancet Respiratory medicine 2013;**1**(4):329-38.
7. Rosenzweig I, Glasser M, Polsek D, et al. Sleep apnoea and the brain: a complex relationship. The Lancet Respiratory medicine 2015;**3**(5):404-14.
8. Sales LV, Bruin VM, D'Almeida V, et al. Cognition and biomarkers of oxidative stress in obstructive sleep apnea. Clinics (Sao Paulo, Brazil) 2013;**68**(4):449-55.
9. Quera Salva MA, Barbot F, Hartley S, et al. Sleep disorders, sleepiness, and near-miss accidents among long-distance highway drivers in the summertime. Sleep medicine 2014;**15**(1):23-6.
10. Stevenson MR, Elkington J, Sharwood L, et al. The role of sleepiness, sleep disorders, and the work environment on heavy-vehicle crashes in 2 Australian states. Am J Epidemiol 2014;**179**(5):594-601.
11. Zhou J, Camacho M, Tang X, et al. A review of neurocognitive function and obstructive sleep apnea with or without daytime sleepiness. Sleep medicine 2016;**23**:99-108.
12. Crawford-Achour E, Dauphinot V, Martin MS, et al. Protective Effect of Long-Term CPAP Therapy on Cognitive Performance in Elderly Patients with Severe OSA: The PROOF Study. Journal of clinical sleep medicine : JCSM : official publication of the American Academy of Sleep Medicine 2015;**11**(5):519-24.
13. Tahmasian M, Rosenzweig I, Eickhoff SB, et al. Structural and functional neural adaptations in obstructive sleep apnea: An activation likelihood estimation meta-analysis. Neuroscience and biobehavioral reviews 2016;**65**:142-56.
14. Henderson LA, Fatouleh RH, Lundblad LC, et al. Effects of 12 Months Continuous Positive Airway Pressure on Sympathetic Activity Related Brainstem Function and Structure in Obstructive Sleep Apnea. Frontiers in neuroscience 2016;**10**:90.
15. Dalmases M, Sole-Padulles C, Torres M, et al. Effect of CPAP on Cognition, Brain Function, and Structure Among Elderly Patients With OSA: A Randomized Pilot Study. Chest 2015;**148**(5):1214-23.
16. Rosenzweig I, Glasser M, Crum WR, et al. Changes in Neurocognitive Architecture in Patients with Obstructive Sleep Apnea Treated with Continuous Positive Airway Pressure. EBioMedicine 2016;**7**:221-9.
17. Rogers GB, Keating DJ, Young RL, et al. From gut dysbiosis to altered brain function and mental illness: mechanisms and pathways. Molecular psychiatry 2016;**21**(6):738-48.
18. Forsythe P, Sudo N, Dinan T, et al. Mood and gut feelings. Brain, behavior, and immunity 2010;**24**(1):9-16.
19. Moreno-Indias I, Torres M, Montserrat JM, et al. Intermittent hypoxia alters gut microbiota diversity in a


For peer review only

mouse model of sleep apnoea. The European respiratory journal 2015;**45**(4):1055-65.

20. Durgan DJ, Ganesh BP, Cope JL, et al. Role of the Gut Microbiome in Obstructive Sleep Apnea-Induced Hypertension. Hypertension 2016;**67**(2):469-74.
21. Chan AW, Tetzlaff JM, Altman DG, et al. SPIRIT 2013 statement: defining standard protocol items for clinical trials. Annals of internal medicine 2013;**158**(3):200-7.
22. Schulz KF, Altman DG, Moher D. CONSORT 2010 statement: updated guidelines for reporting parallel group randomized trials. Annals of internal medicine 2010;**152**(11):726-32.
23. Wu SQ, Liao QC, Xu XX, et al. Effect of CPAP therapy on C-reactive protein and cognitive impairment in patients with obstructive sleep apnea hypopnea syndrome. Sleep & breathing = Schlaf & Atmung 2016.

For peer review only

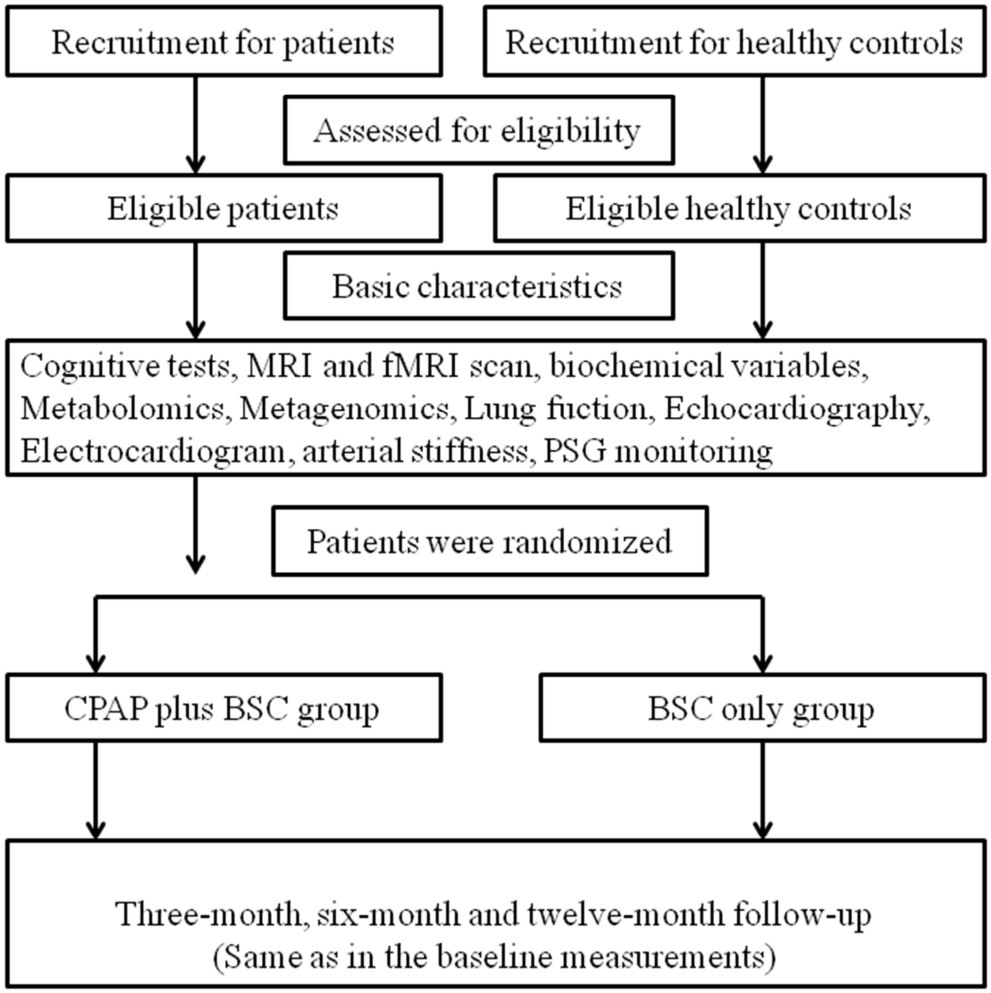


Figure 1. The flow diagram of this RCT

180x190mm (300 x 300 DPI)

BMJ Open

# Effects of Continuous Positive Airway Pressure on Cognitive Function, Neurocognitive Architecture and Function in Patients with Obstructive Sleep Apnea: Study Protocol for A Multicentre, Randomised, Controlled Trial



| | |
|---|---|
| Journal: | *BMJ Open* |
| Manuscript ID | bmjopen-2016-014932.R1 |
| Article Type: | Protocol |
| Date Submitted by the Author: | 11-Jan-2017 |





| | |
|---|---|
| Complete List of Authors: | Xu, Huajun; Shanghai 6th Peoples Hospital Affiliated to Shanghai Jiaotong University School of Medicine, Department of Otolaryngology Head and Neck Surgery & Center of Sleep Medicine<br>Wang, Hui; Shanghai 6th Peoples Hospital Affiliated to Shanghai Jiaotong University School of Medicine, Department of Otolaryngology Head and Neck Surgery & Center of Sleep Medicine<br>Guan, Jian; Shanghai 6th Peoples Hospital Affiliated to Shanghai Jiaotong University School of Medicine, Department of Otolaryngology Head and Neck Surgery & Center of Sleep Medicine<br>Yi, Hongliang; Shanghai 6th Peoples Hospital Affiliated to Shanghai Jiaotong University School of Medicine, Department of Otolaryngology Head and Neck Surgery & Center of Sleep Medicine<br>Qian, Yingjun; Shanghai 6th Peoples Hospital Affiliated to Shanghai Jiaotong University School of Medicine, Department of Otolaryngology Head and Neck Surgery & Center of Sleep Medicine<br>Zou, Jianyin; Shanghai 6th Peoples Hospital Affiliated to Shanghai Jiaotong University School of Medicine, Department of Otolaryngology Head and Neck Surgery & Center of Sleep Medicine<br>Xia, Yunyan; Shanghai 6th Peoples Hospital Affiliated to Shanghai Jiaotong University School of Medicine, Department of Otolaryngology Head and Neck Surgery & Center of Sleep Medicine<br>Fu, Yiqun; Shanghai 6th Peoples Hospital Affiliated to Shanghai Jiaotong University School of Medicine, Department of Otolaryngology Head and Neck Surgery & Center of Sleep Medicine<br>Li, Xinyi; Shanghai 6th Peoples Hospital Affiliated to Shanghai Jiaotong University School of Medicine, Department of Otolaryngology Head and Neck Surgery & Center of Sleep Medicine<br>Jiao, Xiao; Shanghai 6th Peoples Hospital Affiliated to Shanghai Jiaotong University School of Medicine, Department of Otolaryngology Head and Neck Surgery & Center of Sleep Medicine<br>Huang, Hengye; Shanghai Jiao Tong University, Department of Epidemiology, School of Public Health<br>Dong, Pin; Shanghai Jiaotong University First People's Hospital, Department of Otorhinolaryngology<br>YU, Ziwei; Shanghai Jiaotong University First People's Hospital, Department of Otorhinolaryngology<br>Yang, Jun; Shanghai Jiaotong University School of Medicine Xinhua Hospital, Department of Otolaryngology Head and Neck Surgery<br>Xiang, Mingliang; Shanghai Jiaotong University School of Medicine Xinhua |

| | |
|---|---|
| | Hospital, Department of Otolaryngology Head and Neck Surgery<br>Li, Jiping; Shanghai Jiao Tong University School of Medicine Affiliated Renji Hospital, Department of Otolaryngology Head and Neck Surgery<br>Chen, Yanqing; Shanghai Jiao Tong University School of Medicine Affiliated Renji Hospital, Department of Otolaryngology Head and Neck Surgery<br>Wang, Peihua; Shanghai 9th Peoples Hospital Affiliated to Shanghai Jiaotong University School of Medicine, Department of Otolaryngology Head and Neck Surgery<br>Sun, Yizhou; Shanghai 9th Peoples Hospital Affiliated to Shanghai Jiaotong University School of Medicine, Department of Otolaryngology Head and Neck Surgery<br>Li, Yuehua; Shanghai 6th Peoples Hospital Affiliated to Shanghai Jiaotong University School of Medicine, Department of Radiology<br>Zheng, Xiaojian; Shanghai 6th Peoples Hospital Affiliated to Shanghai Jiaotong University School of Medicine, Shanghai Key Laboratory of Diabetes Mellitus and Center for Translational Medicine<br>Jia, Wei; Shanghai 6th Peoples Hospital Affiliated to Shanghai Jiaotong University School of Medicine, Shanghai Key Laboratory of Diabetes Mellitus and Center for Translational Medicine<br>Yin, Shankai; Shanghai 6th Peoples Hospital Affiliated to Shanghai Jiaotong University School of Medicine, Department of Otolaryngology Head and Neck Surgery & Center of Sleep Medicine |



| | |
|---|---|
| <b>Primary Subject Heading</b>: | Ear, nose and throat/otolaryngology |
| Secondary Subject Heading: | Ear, nose and throat/otolaryngology, Neurology |
| Keywords: | obstructive sleep apnea, continuous positive airway pressure, cognitive function, gut microbiota, metabolomics |
| | |


SCHOLARONE™ Manuscripts

For peer review only

# Effects of Continuous Positive Airway Pressure on Cognitive Function, Neurocognitive Architecture and Function in Patients with Obstructive Sleep Apnea: Study Protocol for A Multicentre, Randomised, Controlled Trial

(Running title: Effects of CPAP on Cognitive Function, Neurocognitive Architecture and Function in Patients with OSA)

For peer review only

Xu H MD,Ph D[1,2,3*], Wang H MD,Ph D [1,2,3*], Guan J MD,Ph D [1,2,3], Yi H MD, Ph D [1,2,3], Qian Y MD,Ph D [1,2,3], Zou J MD,Ph D [1,2,3], Xia Y MD [1,2,3], Fu Y MD[1,2,3], Li X MD[1,2,3], Jiao X MD[1,2,3], Huang H MD [4], Dong P MD,Ph D [5], Yu Z, MD,Ph D [5], Yang J MD,Ph D[6], Xiang M MD,Ph D[6], Li J MD,Ph D [7], Chen Y MD,Ph D [7],Wang P MD,Ph D [8], Sun Y, MD,Ph D [8], Li Y MD,Ph D[9], Zheng X,Ph D [10], Jia W MD,Ph D [10,11] , Yin S MD,Ph D [1,2,3]

Both Xu H and Wang H contributed equally to this paper

Corresponding to Guan J & Yin S, guanjian0606@sina.com, skyin@sjtu.edu.cn

1 Department of Otolaryngology Head and Neck Surgery & Center of Sleep Medicine, Shanghai Jiao Tong University Affiliated Sixth People's Hospital, 600 Yishan Road, 200233 Shanghai, China.

2 Otolaryngological Institute of Shanghai Jiao Tong University, 600 Yishan Road, 200233 Shanghai, China.

3 Clinical Research Center, Shanghai Jiao Tong University School of Medicine, 225 South Chongqing Road, 200020 Shanghai, China.

4 Department of Epidemiology, School of Public Health, Shanghai Jiao Tong University, 225South Chongqing Road, 200020 Shanghai, China.

5Department of Otorhinolaryngology, Shanghai First People's Hospital, Shanghai Jiao Tong



For peer review only - http://bmjopen.bmj.com/site/about/guidelines.xhtml

University, 100 Haining Road, 200080 Shanghai, China.

6 Department of Otolaryngology Head and Neck Surgery, Xinhua Hospital, Affiliated to Shanghai Jiaotong University School of Medicine, 1665 Kongjiang Road, 200092 Shanghai, China.

7 Department of Otorhinolaryngology Head and Neck Surgery, Ren Ji Hospital, School of Medicine, Shanghai Jiao Tong University, 160 Pu Jian Road, Shanghai, 200127, China.

8 Department of Otolaryngology Head and Neck Surgery, Shanghai Ninth People's Hospital, Affiliated to Shanghai Jiaotong University School of Medicine, 500 Quxi Road, Shanghai, 200000, China.

9 Department of Radiology, Shanghai Jiao Tong University Affiliated Sixth People's Hospital, 600 Yi Shan Road, Shanghai 200233, China.

10 Shanghai Key Laboratory of Diabetes Mellitus and Center for Translational Medicine, Shanghai Jiao Tong University Affiliated Sixth People's Hospital, , 600 Yishan Road, Shanghai, 200233, China.

11 University of Hawaii Cancer Center, Honolulu, Hawaii, 96813, USA.

For peer review only

## Abstract

### Objectives

Multiple clinical studies have indicated that obstructive sleep apnea (OSA), the most common chronic sleep disorder, may affect neurocognitive function, and that treatment for continuous positive airway pressure (CPAP) has some neurocognitive protective effects against the adverse effects of OSA. However, the effects of CPAP treatment on neurocognitive architecture and function remain unclear. Therefore, this multicenter trial was designed to investigate whether and when neurocognitive architecture and function in patients with OSA can be improved by CPAP treatment, and to explore the role of gut microbiota in improving neurocognitive function during treatment.

### Methods/Design

This study will be a multicenter, randomized, controlled trial with allocation concealment and assessor blinding. A total of 148 eligible patients with moderate to severe OSA will be enrolled from five sleep centers, and randomized to receive CPAP with best supportive care (BSC) intervention or BSC intervention alone. Cognitive function, structure and function of brain regions, gut microbiota, metabolites, biochemical variables, electrocardiography, echocardiography, pulmonary function, and arterial stiffness will be assessed at baseline before randomization and at 3, 6, and 12 months.

### Ethics and Dissemination

Ethics approval was given by the Medical Ethics Committee of Shanghai Jiao Tong University Affiliated Sixth People's Hospital (approval number 2015-79). The findings from this study will be disseminated in peer-reviewed journals and at conferences.

**Trial registration number:** NCT02886156; Pre-results.




For peer review only - http://bmjopen.bmj.com/site/about/guidelines.xhtml

**Keywords:** obstructive sleep apnea, continuous positive airway pressure, cognitive function, gut microbiota, metabolomics

**Article summary**

Strengths and limitations of this study:

1) A multicentric, randomized, prospective study.

2) Relatively large number of participants (n=148).

3) Strictly standardized study population.

4) The multicentre pragmatic design strengthens future results and generalisability.

5) Possible selection bias may be existed as not all possible patients may enter in the study.

For peer review only

**Background**

Obstructive sleep apnea (OSA) is a common chronic sleep disorder characterized by recurrent partial or complete pharyngeal obstruction during sleep [1]. There is a high prevalence (2–4%) of this condition in adults aged 30–60 years, which is increasing with the growing epidemic of obesity [2-4]. OSA is well recognized as a risk factor for neurocognitive, cardiovascular, and metabolic disorders [5-7]. It is worth noting that neurocognitive dysfunction is associated with a series of sequelae, such as social and economic burdens [8-10]. Continuous positive airway pressure (CPAP) is typically recommended as first-line treatment for OSA, as it can partly or completely reverse OSA-related complications, including cognitive impairments [11 12]. In addition, CPAP may be effective for reversing structural and functional brain changes [13 14].

To date, there have only been two randomized controlled trials (RCTs) that simultaneously evaluated the effects of CPAP on cognition and neuroimaging; both studies had relatively small sample sizes and short intervention periods [15 16]. However, the response to CPAP treatment in the trials was variable. Thus, the relationships among OSA, brain, and neurobehavioral function are complex and must be comprehensively evaluated, including how, when, and how long CPAP treatment should be applied for effective therapy.

The gut microbiota is an important factor that affects neurological functions [17], and may be involved in the modulation of central nervous function (e.g., the hypothalamus–pituitary–adrenal [HPA] axis) and associated with neuropsychiatric conditions [17 18]. The composition of the gut microbiota is altered through intermittent hypoxia or sleep fragmentation in animal models [19 20]. However, there have been no studies to determine whether changes in human intestinal microbiota are associated with OSA, and whether or how the microbiota can be modulated by CPAP. This study will clarify the complex


For peer review only - http://bmjopen.bmj.com/site/about/guidelines.xhtml

relationships among OSA, gut microbiota, and neurological functions.

## Methods/Design

### *Study aims and hypotheses*

The primary aim of this study is to assess the dynamic (3, 6, and 12 months) effects of CPAP on cognitive function, neurocognitive architecture and function. The primary hypothesis of this study is that, compared with the non-CPAP treatment group, patients with OSA who receive CPAP intervention will show improved cognitive function through structural and functional brain modulation immediately after 3 months of CPAP treatment and that these benefits will continue at the 6-month and 12-month follow-ups. The secondary aims of this study are to investigate whether and which sleep parameters are correlated with improvements of cognitive function, neurocognitive architecture and function; to explore whether the metabolic status assessed by metabolomics can be reversed by CPAP and correlated with improvement of neurocognitive impairment; and to investigate whether CPAP modulates the gut microbiota and contributes to alleviation of neurocognitive impairment. We hypothesize that patients with OSA who undergo CPAP treatment will have metabolic improvement and modulation of gut microbiota, and that these changes will have beneficial effects on neurocognitive impairment.

### *Study design*

The Shanghai Multicenter Obstructive Sleep Apnea Therapy trial is a multicenter, evaluator-blinded, randomized, parallel controlled trial performed in five sleep centers (Department of Otolaryngology & Head and Neck Surgery, Shanghai Jiao Tong University Affiliated Sixth People’s Hospital; Department of Otolaryngology-Head and Neck Surgery,


For peer review only - http://bmjopen.bmj.com/site/about/guidelines.xhtml

Shanghai First People's Hospital, Shanghai Jiao Tong University; Department of Otolaryngology, Renji Hospital, School of Medicine, Shanghai Jiaotong University; Department of Otolaryngology, Head and Neck Surgery, Xinhua Hospital, Shanghai Jiaotong University School of Medicine; Department of Otolaryngology, The Ninth People's Hospital Affiliated to Shanghai Jiao-Tong University School of Medicine) in the Shanghai district. The design of this protocol strictly followed the Standard Protocol Items Recommendations for Interventional Trials [21] and the Consolidated Standards of Reporting Trials statements [22]. A total of 148 eligible participants with moderate to severe OSA will be enrolled from the abovementioned five sleep centers and randomized to receive CPAP with best supportive care (BSC) intervention or BSC intervention alone. Cognitive function, structure and function of brain regions, metabolites, gut microbiota, biochemical variables, electrocardiography, echocardiography, pulmonary function, and arterial stiffness will be assessed at baseline before randomization and at 3, 6, and 12 months. The flow diagram of this RCT is presented in Figure 1.

**Study population**

The study population (30–65 years old) will be enrolled from Shanghai and the surrounding area, and all of the participants will be from the Han population. The diagnostic, inclusion, and exclusion criteria for this trial will be as described below.

*Diagnostic criteria*

According to the American Academic Sleep Medicine (AASM) 2007 criteria [23], moderate, severe OSA are defined as an apnea-hypopnea index (AHI) $\geq 15$ and 30 events per hour respectively, and the normal state is defined as AHI $< 5$ events per hour.



For peer review only - http://bmjopen.bmj.com/site/about/guidelines.xhtml

*Eligible participants*

*Inclusion criteria*

1) Informed consent
2) Age 30–65 years
3) Newly diagnosed OSA (full-night in-laboratory polysomnography [PSG] with AHI ≥ 15 events per hour)
4) Be Adherent to CPAP treatment
5) No participation in any other clinical trial in the past 3 months
6) Able to accomplish relevant tests and follow-up

*Exclusion criteria*

1) Severe systemic diseases (e.g., cardiac, hepatic, and renal failure)
2) Psychiatric conditions (e.g., depression, mania, schizophrenia)
3) Neurological diseases (e.g., head trauma, brain tumor, epilepsy, stroke, transient ischemic attack, coma)
4) Sleep disorders other than OSA (narcolepsy, insomnia, chronic sleep deprivation, rapid eye movement [REM] behavior disorder and restless legs syndrome, central sleep apnea or obesity hypoventilation syndrome)
5) Alcoholism, drug addiction, use of psychotropic drugs, sedatives, or narcotics
6) Prior therapy for OSA (i.e., CPAP, upper airway surgery, oral appliance)
7) Mini Mental State Examination (MMSE)< 24
8) Left-handed
9) MRI contraindications (e.g., claustrophobic or metal implantation)
10) Gastrointestinal surgery during the last year, except for appendicitis and hernia surgery

For peer review only

11) Pregnancy

12) Use of intestinal flora regulator (e.g., antibiotics or probiotics) in the previous 8 weeks

13) Medical treatment for cholecystitis, gallstones, gastrointestinal ulcers, urinary tract infection, acute pyelonephritis, or cystitis in the past 3 months

14) Infectious diseases, such as tuberculosis, acquired immune deficiency syndrome

15) Deemed by the researchers to be unsuitable for this trial

**Withdrawal criteria and management**

Subjects will be allowed or required to withdraw from this trial for insurmountable claustrophobia or other unbearable conditions of CPAP intolerance, major protocol violation, or the development of serious disease that prevents continuation in the trial.

**Recruitment and screening**

Participants will be recruited from Shanghai and its vicinity through newspapers and instant messaging software (WeChat). Potential participants with self-reported severe snoring and with an interest in participating will be screened by a standard PSG and examined for eligibility (AHI $\geq$ 15 times per hour) for multicenter RCT. Eligible participants will be given information about this trial and have an informed discussion with trained research assistants regarding the information provided. Written informed consent will be obtained from each participant prior to enrollment.

**Randomization, allocation concealment, and blinding**

After baseline assessment, eligible patients with moderate to severe OSA will be randomly and equally allocated (the randomization is 1:1 ratio) to the CPAP plus BSC group or BSC only group. The central random allocation sequence will be produced using the statistical



For peer review only - http://bmjopen.bmj.com/site/about/guidelines.xhtml

software SAS v.9.4 and managed by an independent statistician (from Department of Epidemiology, School of Public Health, Shanghai Jiao Tong University) blinded to the recruitment, evaluation, and intervention of the participants. The research assistant from each sleep center will request (via E-mail) a participant's allocation from the statistician. The group allocation will not be masked by participants or intervention supervisors in this trial; however, it can be blinded to outcome assessors and data statisticians, they will be blinded to the patients' group assignments (i.e., nobody will tell them which patient is allocated to CPAP treatment group or BSC group).

**Intervention**

*BSC only group*

Participants in the BSC only group will receive advice regarding lifestyle modification, sleep hygiene, naps, exercise, caffeine, and diet, and avoiding alcohol consumption, but no specific weight loss program, diet, or salt restriction will be suggested.

*CPAP plus BSC group*

Participants in the CPAP plus BSC group will receive CPAP treatment plus the aforementioned BSC intervention. CPAP treatment (LOTUS AUTO; Curative Medical Technology Inc., Beijing, China) will be initiated using standard clinical practice (before CPAP treatment, the standard clinical practice is the doctors will teach the patients how to use the CPAP machine and comfort them) at each center. Through a chip in this machine, we can monitor usage of CPAP from every participant in our computer terminal through wireless network and might be helpful for improving compliance of CPAP by guidance and assistance. Participants in both groups will be asked to continue their usual medical care during the trial.



For peer review only - http://bmjopen.bmj.com/site/about/guidelines.xhtml

For peer review only

## Follow-up

At 3, 6, and 12 months during the intervention period, all of the participants will undergo the same examinations as at baseline. During the follow-up period, CPAP usage information will be automatically downloaded daily from auto CPAP via the Internet.

## Outcome assessment

The variables in this trial consist of basic characteristics, primary outcomes, and secondary outcomes. The basic characteristics will be measured at baseline, and primary and secondary outcomes will be measured at baseline and at the end of each intervention period (3, 6, and 12 months after randomization). All of the primary and secondary outcomes will be assessed by experienced staff at each sleep center who will be blinded to treatment allocation.

## Basic characteristics

Participants' demographic characteristics (e.g., age, sex, years of education, medical history, medication use, occupation) will be collected by recruiters using a questionnaire. The basic Montreal Cognitive Assessment (MoCA) and MMSE scores will be assessed using the corresponding standardized scales. The assessors in each sleep center will be trained by a neurologist. Baseline assessment will be completed before randomization.

## Primary and secondary outcomes

*Primary endpoints*

MoCA

*Secondary endpoints*

MMSE

Default mode network (DMN) and cortical thickness (CTh) measured by functional and

For peer review only

structural magnetic resonance imaging (MRI).

Cerebral metabolites, such as N-acetylaspartate (NAA), choline (Cho), creatine (Cr), glutamate and glutamine (Glx), and myo-inositol (mI) measured by magnetic resonance spectroscopy (MRS).

Neuropsychological tests includes mental arithmetic, memory scanning, movement perception, switching attention, space location memory span, attention allocation choice reaction time curve fit will be also performed.

Sleep variables (apnea-hypopnea index [AHI], oxygen desaturation index [ODI], lowest oxygen saturation [$LSaO_2$]) mean oxygen saturation [mean $SaO_2$], micro-arousal index [MAI], total sleep time, total REM sleep time and total slow wave sleep time, etc.).

Auditory Testing

Central auditory processing testing (Speech-in-noise test, Competing sentences test, Gap detection, Dichotic listening)

256-channel high-density EEG recordings

Microbial community profiling in the gut will be quantified by metagenomics analysis [24].

Low-molecular-weight metabolites( < 1000 KDa) will be quantified by metabolomics analysis [25].

Amyloid β protein 42, pre-senilin 1, amyloid-β protein 40, total GSK-3β, and serine-9 phosphorylated GSK-3β

*Other outcomes*

Biochemical variables: lipid profile (total cholesterol, triglycerides, high-density lipoprotein cholesterol, low-density lipoprotein cholesterol, apoA-I, apoB, apoE, and lipoprotein(a)), glucose, insulin, HbA1c, free fatty acids, cholinesterase, lipopolysaccharide binding protein,

For peer review only

toll-like receptor-4, TLR-2, hepatic function tests (alanine aminotransferase, aspartate aminotransferase, γ-glutamyl endopeptidase, alkaline phosphatase, total protein, albumin, total bilirubin, direct bilirubin, bile acid, prealbumin), renal function tests (creatinine, urea nitrogen, uric acid), microalbuminuria; apolipoprotein E genotyping

Inflammatory biomarkers: leptin, RANTES, monocyte chemotactic factor 1, interleukin (IL)-6, IL-8, tumor necrosis factor-α, hypersensitive C-reactive protein, resistin, serum amyloid A protein, α-acid glycoprotein

Anti-inflammatory biomarkers: adiponectin, IL-1Ra

Oxidative stress index: 8-isoprostane

Blood pressure (systolic blood pressure, diastolic blood pressure)

Anthropometric variables (weight, height, waist, hip, chest and neck circumferences, visceral fat area, subcutaneous fat area)

Echocardiography

Arterial stiffness (pulse wave velocity, ankle brachial index, toe-brachial index)

Lung function (e.g., forced expiratory volume in 1 s, forced vital capacity)

Electrocardiography (ECG)

**Study examination procedures**

All of the study measurements will be performed before and after 3, 6, and 12 months of CPAP intervention at each center except for lung function and ECG.

*Anthropometric measurements*: Height and weight will be measured using standard methods. BMI (weight [kg]/height$^2$[m$^2$]) will be calculated from height and weight. Waist, hip, and neck circumferences will be measured using a measuring tape in the conventional manner.

*Urine samples*: First midstream urine samples will be collected in the morning with the


For peer review only - http://bmjopen.bmj.com/site/about/guidelines.xhtml

collected urine samples stored immediately at –80°C for further analyses.

*Blood samples*: Fasting blood samples will be collected in the morning, and the samples will be immediately sent for further analyses such as biochemical analysis.

*Fecal samples*: Fecal samples will be collected and stored at –80°C until use in subsequent assays. The composition of microbiota will be measured using 16S rRNA-hybridization and DNA staining.

*Sleep respiratory variables*: Laboratory-based PSG (Alice 4 or 5, Respironics; Pittsburgh, PA, USA) will be used to record sleep parameters. Electroencephalography (EEG), electrooculography, ECG, electromyography, nasal/oral airflow, thoracic/abdominal movement, pulse oximetry, body posture, and snoring will be recorded. The definition of OSA is established according to the AASM 2007 criteria.

*Neuropsychological tests*: Two questionnaires (MoCA and MMSE) will be administered. The neuropsychological tests includes mental arithmetic, memory scanning, movement perception, switching attention, space location memory span, attention allocation choice reaction time curve fit will be also performed.

*Auditory testing*: Audiograms will be measured in one-octave steps at frequencies ranging from 125 Hz to 8 kHz and then at 10, 12.5, 16, and 20 kHz. DPOAE values will be recorded in both groups using a Madsen Capella cochlear emissions analyzer in a soundproof room. Only patients with normal audiometry (threshold below 25 dB HL at all frequencies from 0.125 to 20 kHz), normal DPOAE levels, normal tympanometric curve of type A, and presence of ipsi- and contralateral stapedial reflexes will be included.

*Central auditory processing testing*: The following central auditory processing tests will be performed in a soundproof room. Before the formal test, participants will be allowed to

For peer review only

practice as many times as required and provide feedback to become familiar with the stimuli. (1) Speech-in-noise test: All of the signal processing will be performed with MATLAB software. The speech of 20 ten-word sentences will be presented under both quiet and noisy conditions with speech-shaped noise, and will be delivered bilaterally through Sennheiser HD205 headphones at a signal-to-noise ratio of –5 dB. (2) Competing sentences test: Babble noise and the target sentence will be presented bilaterally at an intensity level of 65 dB SL (above speech reception threshold) at a signal-to-noise ratio of –3 dB. (3) Gap detection: The purpose of this evaluation is to determine the smallest interval that the listener can detect. Two stimuli separated in time by a “gap” will be presented and the listener will be asked to detect the gap, essentially discriminating sound from silence. Stimuli as white noise will be bilaterally presented as background noise at intensities 40 dB higher than the hearing threshold. (4) Dichotic listening: Two different numbers will be simultaneously presented to both ears with the subjects instructed to repeat both numbers in any order. The test includes 20 number pairs presented dichotically. The number pairs will be aligned for onset and offset to eliminate any cue for the first number heard. Dichotic listening performance will be scored as the percentage of correctly reported different numbers from the right and left ears in the noise-exposed and control groups.

*256-Channel high-density EEG recording*: EEG data will be recorded with 256 channels on EGI’s HydroCel Geodesic Sensor Net, with Cz used as the reference channel. All of the electrode impedances will be detected and maintained at less than 50 kΩ. The oddball stimuli pattern with speech sound is used in this study (via Eprime 2.0; Psychology Software Tools). The auditory oddball task includes presentation sound of /Ba/, whereas /Da/ is presented as the “oddball” target stimulus. The stimulus /Ba/ is presented in 85% of the trials together with



For peer review only - http://bmjopen.bmj.com/site/about/guidelines.xhtml

For peer review only

the stimulus /Da/ in 15% of the trials. The whole task consists of a total of 1000 auditory stimuli with random interstimulus intervals ranging from 850 ms to 1450 ms. The sound stimuli with an intensity of 75 dB delivered through two loudspeakers at a distance of 100 cm from the subjects. During the P3 cortical waves test, the subjects will be asked to press a switch held in the right hand each time they detect a target tone with the eyes closed. In addition, they will be asked not to respond to any auditory stimulus while watching silent films during the MMN test. Source analysis of event-related potentials as MMN, describing neural sources of measured scalp potentials, will be estimated with GeoSource (version 2.0).

*Brain imaging:* All the MRI scans will be performed on a Siemens 3T Verio scanner (MAGETOM, Siemens) using a 12-channel coil using the same parameters. A high-resolution T1-3D, T2-3D weighted sequences MRI scan will be conducted for all the subjects. T1 3D axial images: FOV, 230 mm; TR/TE, 1500/2.96 ms; flip angle, 9°; voxel size, $0.9\times0.9\times1mm^3$; slice thickness, 1 mm; and distance factor, 50%. T2 3D axial images: FOV, 230 mm; TR/TE,1500/2.96 ms; voxel size, $0.9\times0.9\times1mm^3$; slice thickness, 1 mm; and distance factor, 50%. At the same position, rs-fMRI BOLD sequence acquired during the rest condition. 240 functional images will be obtained (repetition time 2,000 m sec, echo time 30 m sec, thickness 4.0 mm, gap 1.2 mm, acquisition matrix 64×64, flip angle 90 degrees, field of view 230 mm ×230 mm, 30 axial slices with gradient-recalled echo-planar imaging pulse sequence covering the whole brain). Patients will be asked to keep their eyes closed and not to focus their thoughts on any particular topic and not to fall asleep. DKI will be performed in all subjects using 30 gradient directions, six b values and an echo-planar DWI sequence. FOV, 300 mm; TR/TE, 1100/109 ms; matrix size 128×128; and slice thickness: 4 mm.

*Cerebral metabolites*: MRS scans will be performed on a Siemens 3T Verio scanner using a

For peer review only

12-channel coil. A 2.0×2.0×2.0 $cm^3$ voxel of interest (VOI) will be placed at the frontal lobe. NAA, Cho, and Cr will be acquired by a MEGA-PRESS sequence with the following parameters: TE/TR=135/5000 ms; number of average =80 (scan time= 8 min).

*Abdominal and liver fat measurements:* The hepatic, subcutaneous fat and visceral fat will be measured through axial scanning in upper abdomen (from diaphragm to the lower edge of liver). The participants should hold to breath in order to reduce the artifacts induced by respiratory movement. Anatomical T1-weighted spin-echo MR images will be obtained using the following parameters: slice thickness = 5 mm; slice spacing = 2.5 mm.

**Safety measurements**

Any unexpected adverse events during the intervention period will be monitored, reported to a research assistant, and causality in relation to CPAP intervention and the severity of adverse events will be analyzed. Data we submit will be monitored by Ethics Committee of Shanghai Jiao Tong University Affiliated Sixth People's Hospital. Serious adverse events will be immediately reported to the ethics committee.

**Sample size calculation**

A previous study indicated that the mean and standard deviation (SD) of MoCA score are 26.1 (2.0) and 27.4 (2.3) before and after CPAP treatment, respectively [26]. With a significance level of 0.05 and statistical power of 0.9, the calculated sample size is 59 for each group. Considering a 20% loss to follow-up, a total of 148 participants will be required for randomization.

**Statistical methods**

Study analysis will be performed by intention-to-treat (ITT). All of the statistical analyses


For peer review only - http://bmjopen.bmj.com/site/about/guidelines.xhtml

will be performed using SAS v.9.4 software (SAS Institute, Cary, North Carolina, USA), with a two-sided p value < 0.05 indicating statistical significance. The Kolmogorov–Smirnov test will be performed to test normality of the distribution. Continuous variables will be expressed as means and SD or median (first third quartile) for continuous variables and percentages for categorical variables. To assess primary and secondary outcomes, the mixed-effects linear model with restricted maximum likelihood will be used to analyze the effect of CPAP treatment on the continuous variables, and logistic regression models for dependent categorical variables. In each model, the independent variables are group (CPAP plus BSC group, BSC group), different measurement time (e.g., baseline, 3, 6 and 12-month follow-up), confounding factors (e.g., age, BMI) and any inequality factors among groups at the baseline assessment. The post hoc comparison between groups at different time points will be performed using multiple comparisons. A multiple imputation method will be used to fill in missing data.

**Data collection and management**

All of the data collectors and evaluators will be blinded to the group assignments. The data in this trial will be collected using print-based case report forms, which will be transcribed by a research assistant into web-based case report forms (w-CRFs) with an electronic data capture system (EDC). The EDC will be provided by the School of Public Health of Shanghai Jiao Tong University (Shanghai, China) and will meet the available standards of security. Data in w-CRFs will be stored in the EDC system at separate password-protected locations. The research assistants will be responsible for ensuring the integrity and accuracy of all data when data are transcribed into w-CRFs by checks on value ranges and logical checks. Development and validation of the study database, quality control, and extraction of data will be performed


For peer review only - http://bmjopen.bmj.com/site/about/guidelines.xhtml

according to the procedures of the medical research platform.

**Ethical issues**

This protocol will be conducted in accordance with the Declaration of Helsinki. Ethics approval has been obtained from Shanghai Jiao Tong University Affiliated Sixth People's Hospital (Ethics number: 2015–79). Written informed consent will be obtained from each participant with a full discussion about the potential benefits and risks before participation.

**Dissemination**

This study will be published in scientific journals and presented at national conferences in the field of neurology and sleep medicine. Study results will also be sent to study participants and disseminated to researchers, healthcare providers, healthcare professionals, and the general public regardless of the magnitude or direction of effects.

**Discussion**

Increasing numbers of clinical studies have shown that CPAP is an effective therapeutic method for neurocognitive dysfunction in OSA. However, the effects and duration of CPAP treatment on neurocognitive function have not been clearly established. This study focuses on the effects of CPAP on middle-aged patients with moderate to severe OSA. Through a 3-, 6-, and 12-month intervention with CPAP treatment, the results from a range of clinical and biochemical assessments will provide clearer information on the effects of treatment in OSA.

Measurements of neurocognitive function before and after intervention in this trial may strengthen evidence for links between OSA and neurocognitive dysfunction, or reveal new associations. Comparing patients with OSA to healthy controls will allow us to identify differences in neurocognitive function between the two groups. In addition, through CPAP

For peer review only

intervention, it will be possible to explore the mechanism of action of CPAP on neurocognitive function (including structural and functional effects), allowing us to investigate the causal relationships between OSA and neurocognitive dysfunction.

The microbial community structure in rodent animals was changed when the rodents were under intermittent hypoxia or sleep fragmentation status [19 20 27]. In addition, sleep fragmentation-induced metabolic alterations may be mediated by concurrent changes in gut microbiota [27]. Thus, gut microbiome-targeted therapeutics may help to reduce the major end-organ morbidities of OSA. Through metagenomics and metabolomics approaches, we will determine whether gut microbiota play a pivotal role in OSA-related complications, especially in alleviating neurocognitive dysfunction.

To the best of our knowledge, the proposed study will be the first to investigate the effects of CPAP on neurocognitive dysfunction in OSA and to explore the underlying mechanisms using combined "-omics" approaches. The results of this multicenter trial may be helpful for improving the neurocognitive function of patients with OSA.

This protocol describes a multicenter RCT to assess the effects of CPAP treatment on neurocognitive function in patients with moderate to severe OSA. To reduce potential bias, multiple rigorous methods, such as randomization, blinding of raters and statistical analyzers, and ITT principles, will be implemented. Potential limitations of this protocol should also be addressed. First, sham CPAP is not used as a placebo; therefore neither participants nor trial researchers will be blinded to the protocol. Thus, performance bias may be inevitable. Second, although all of the raters have been trained by the same neurologist, there may be heterogeneity in neuropsychological tests in each center.

In summary, this will be the first multicenter RCT to dynamically observe the effects of

For peer review only



For peer review only - http://bmjopen.bmj.com/site/about/guidelines.xhtml

CPAP on neurocognitive function and to explore the role of the gut microbiota in neurocognitive dysfunction in OSA. The results of this study will help to clarify potential mechanisms for alleviation of OSA and improvement of neurocognitive dysfunction.

**Trial status:** At the time of manuscript submission, the enrollment of volunteers is ongoing.

**Acknowledgements** This study was supported by grants-in-aid from multi-center clinical research project from school of medicine, Shanghai Jiao Tong University (DLY201502). The study sponsor played no role in the design, methods, data management or analysis or in the decision to publish. The corresponding authors have full access to all the data in the study and have final responsibility for the decision to submit for publication.

**Contributors** Xu H, Wang H and Yin S conceived the study, designed the study protocol, and drafted the manuscript. Xu H, Wang H, Guan J, Yi H, Qian Y, Zou J, Xia Y, Fu Y, Li X, Jiao X, Dong P, Yu Z, Yang J, Xiang M, Li J, Chen Y, Wang P, Sun Y, Li Y, Jia W and Yin S participated in the coordination and implementation of the study. Xu H, Wang H and Yin S revised and finalized the study protocol. Yin S is in charge of coordination and direct implementation. Huang H helped to develop the study measures and analyses. All authors contributed to drafting the manuscript and have read and approved the final manuscript.

**Competing interests** The authors declare that they have no competing interests.

**Patient consent** Obtained.

**Ethics approval** Obtained.

**References:**

1. Strollo PJ, Jr., Rogers RM. Obstructive sleep apnea. *The New England journal of medicine* 1996;334(2):99-104. doi: 10.1056/nejm199601113340207 [published Online First: 1996/01/11]

2. Young T, Palta M, Dempsey J, et al. The occurrence of sleep-disordered breathing among middle-aged adults. *The New England journal of medicine* 1993;328(17):1230-5. doi: 10.1056/nejm199304293281704 [published Online First: 1993/04/29]

3. Peppard PE, Young T, Barnet JH, et al. Increased prevalence of sleep-disordered breathing in adults. *Am J Epidemiol* 2013;177(9):1006-14. doi: 10.1093/aje/kws342 [published Online First: 2013/04/17]



For peer review only - http://bmjopen.bmj.com/site/about/guidelines.xhtml

4. Heinzer R, Vat S, Marques-Vidal P, et al. Prevalence of sleep-disordered breathing in the general population: the HypnoLaus study. *The Lancet Respiratory medicine* 2015;3(4):310-8. doi: 10.1016/s2213-2600(15)00043-0 [published Online First: 2015/02/16]

5. Sanchez-de-la-Torre M, Campos-Rodriguez F, Barbe F. Obstructive sleep apnoea and cardiovascular disease. *The Lancet Respiratory medicine* 2013;1(1):61-72. doi: 10.1016/s2213-2600(12)70051-6 [published Online First: 2013/12/11]

6. Aurora RN, Punjabi NM. Obstructive sleep apnoea and type 2 diabetes mellitus: a bidirectional association. *The Lancet Respiratory medicine* 2013;1(4):329-38. doi: 10.1016/s2213-2600(13)70039-0 [published Online First: 2014/01/17]

7. Rosenzweig I, Glasser M, Polsek D, et al. Sleep apnoea and the brain: a complex relationship. *The Lancet Respiratory medicine* 2015;3(5):404-14. doi: 10.1016/s2213-2600(15)00090-9 [published Online First: 2015/04/19]

8. Sales LV, Bruin VM, D'Almeida V, et al. Cognition and biomarkers of oxidative stress in obstructive sleep apnea. *Clinics (Sao Paulo, Brazil)* 2013;68(4):449-55. doi: 10.6061/clinics/2013(04)03 [published Online First: 2013/06/20]

9. Quera Salva MA, Barbot F, Hartley S, et al. Sleep disorders, sleepiness, and near-miss accidents among long-distance highway drivers in the summertime. *Sleep medicine* 2014;15(1):23-6. doi: 10.1016/j.sleep.2013.06.018 [published Online First: 2013/11/30]

10. Stevenson MR, Elkington J, Sharwood L, et al. The role of sleepiness, sleep disorders, and the work environment on heavy-vehicle crashes in 2 Australian states. *Am J Epidemiol* 2014;179(5):594-601. doi: 10.1093/aje/kwt305 [published Online First: 2013/12/20]

11. Zhou J, Camacho M, Tang X, et al. A review of neurocognitive function and obstructive sleep apnea with or without daytime sleepiness. *Sleep medicine* 2016;23:99-108. doi: 10.1016/j.sleep.2016.02.008 [published Online First: 2016/06/12]

12. Crawford-Achour E, Dauphinot V, Martin MS, et al. Protective Effect of Long-Term CPAP Therapy on Cognitive Performance in Elderly Patients with Severe OSA: The PROOF Study. *Journal of clinical sleep medicine : JCSM : official publication of the American Academy of Sleep Medicine* 2015;11(5):519-24. doi: 10.5664/jcsm.4694 [published Online First: 2015/02/24]

13. Tahmasian M, Rosenzweig I, Eickhoff SB, et al. Structural and functional neural adaptations in obstructive sleep apnea: An activation likelihood estimation meta-analysis. *Neuroscience and biobehavioral reviews* 2016;65:142-56. doi: 10.1016/j.neubiorev.2016.03.026 [published Online First: 2016/04/04]

14. Henderson LA, Fatouleh RH, Lundblad LC, et al. Effects of 12 Months Continuous Positive Airway Pressure on Sympathetic Activity Related Brainstem Function and Structure in Obstructive Sleep Apnea. *Frontiers in neuroscience* 2016;10:90. doi: 10.3389/fnins.2016.00090 [published Online First: 2016/03/26]

15. Dalmases M, Sole-Padulles C, Torres M, et al. Effect of CPAP on Cognition, Brain Function, and Structure Among Elderly Patients With OSA: A Randomized Pilot Study. *Chest* 2015;148(5):1214-23. doi: 10.1378/chest.15-0171 [published Online First: 2015/06/13]

16. Rosenzweig I, Glasser M, Crum WR, et al. Changes in Neurocognitive Architecture in Patients with Obstructive Sleep Apnea Treated with Continuous Positive Airway Pressure. *EBioMedicine* 2016;7:221-9. doi: 10.1016/j.ebiom.2016.03.020 [published Online First: 2016/06/21]

17. Rogers GB, Keating DJ, Young RL, et al. From gut dysbiosis to altered brain function and mental illness: mechanisms and pathways. *Molecular psychiatry* 2016;21(6):738-48. doi: 10.1038/mp.2016.50 [published Online First: 2016/04/20]

18. Forsythe P, Sudo N, Dinan T, et al. Mood and gut feelings. *Brain, behavior, and immunity* 2010;24(1):9-16. doi: 10.1016/j.bbi.2009.05.058 [published Online First: 2009/06/02]

19. Moreno-Indias I, Torres M, Montserrat JM, et al. Intermittent hypoxia alters gut microbiota diversity in a



For peer review only - http://bmjopen.bmj.com/site/about/guidelines.xhtml

mouse model of sleep apnoea. *The European respiratory journal* 2015;45(4):1055-65. doi: 10.1183/09031936.00184314 [published Online First: 2014/12/30]

20. Durgan DJ, Ganesh BP, Cope JL, et al. Role of the Gut Microbiome in Obstructive Sleep Apnea-Induced Hypertension. *Hypertension* 2016;67(2):469-74. doi: 10.1161/hypertensionaha.115.06672 [published Online First: 2015/12/30]

21. Chan AW, Tetzlaff JM, Altman DG, et al. SPIRIT 2013 statement: defining standard protocol items for clinical trials. *Annals of internal medicine* 2013;158(3):200-7. doi: 10.7326/0003-4819-158-3-201302050-00583 [published Online First: 2013/01/09]

22. Schulz KF, Altman DG, Moher D. CONSORT 2010 statement: updated guidelines for reporting parallel group randomized trials. *Annals of internal medicine* 2010;152(11):726-32. doi: 10.7326/0003-4819-152-11-201006010-00232 [published Online First: 2010/03/26]

23. Iber C A-IS, Chesson AL, Jr., Quan SF. for the American Academy of Sleep Medicine. The AASM manual for the scoring of sleep and associated events: rules, terminology and technical specifications. 1st ed. Westchester, IL: American Academy of Sleep Medicine. 2007

24. Zhang C, Yin A, Li H, et al. Dietary Modulation of Gut Microbiota Contributes to Alleviation of Both Genetic and Simple Obesity in Children. *EBioMedicine* 2015;2(8):968-84. doi: 10.1016/j.ebiom.2015.07.007 [published Online First: 2015/10/02]

25. Xu H, Zheng X, Qian Y, et al. Metabolomics Profiling for Obstructive Sleep Apnea and Simple Snorers. *Scientific reports* 2016;6:30958. doi: 10.1038/srep30958 [published Online First: 2016/08/03]

26. Wu SQ, Liao QC, Xu XX, et al. Effect of CPAP therapy on C-reactive protein and cognitive impairment in patients with obstructive sleep apnea hypopnea syndrome. *Sleep & breathing = Schlaf & Atmung* 2016 doi: 10.1007/s11325-016-1331-2 [published Online First: 2016/03/20]

27. Poroyko VA, Carreras A, Khalyfa A, et al. Chronic Sleep Disruption Alters Gut Microbiota, Induces Systemic and Adipose Tissue Inflammation and Insulin Resistance in Mice. *Scientific reports* 2016;6:35405. doi: 10.1038/srep35405 [published Online First: 2016/10/16]

For peer review only

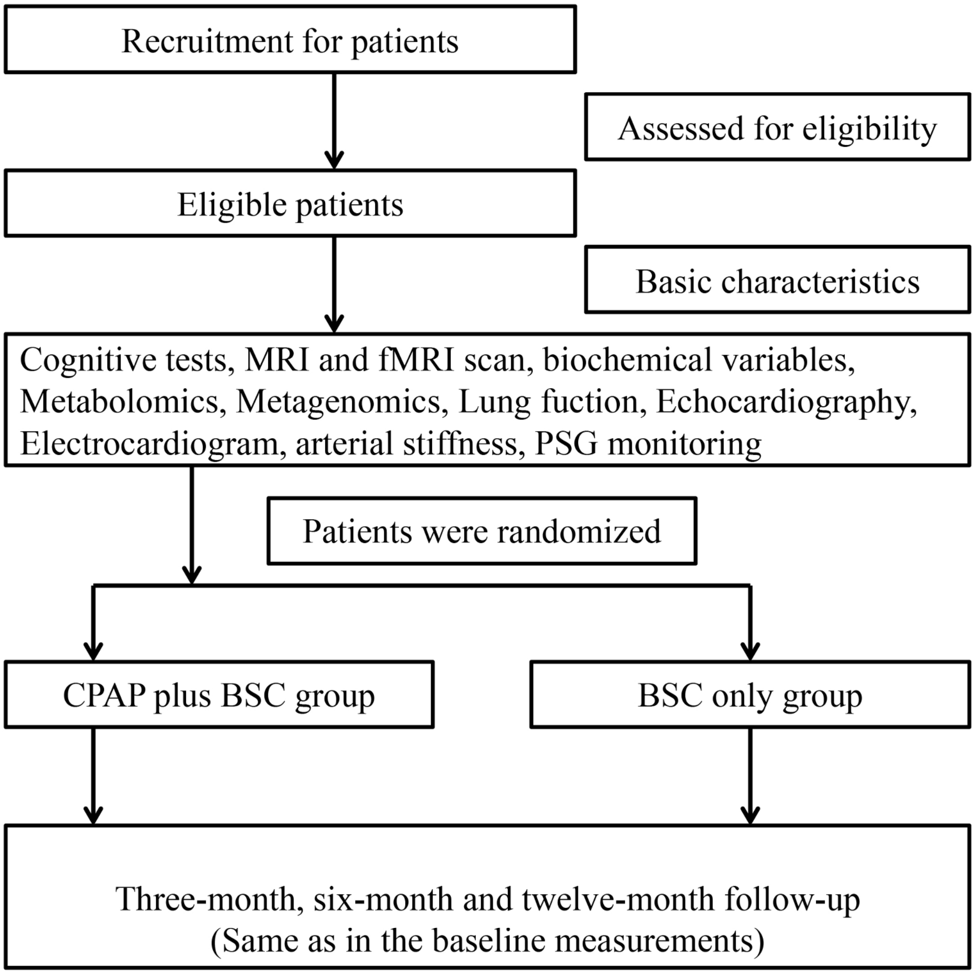


Figure 1. The flow diagram of this RCT
Figure 1
182x190mm (300 x 300 DPI)

STANDARD PROTOCOL ITEMS: RECOMMENDATIONS FOR INTERVENTIONAL TRIALS

SPIRIT 2013 Checklist: Recommended items to address in a clinical trial protocol and related documents*

| Section/item | Item No | Description | |
|---|---|---|---|
| **Administrative information** | | | |
| Title | 1 | Descriptive title identifying the study design, population, interventions, and, if applicable, trial acronym | 1 |
| Trial registration | 2a | Trial identifier and registry name. If not yet registered, name of intended registry | 3 |
| | 2b | All items from the World Health Organization Trial Registration Data Set | 3 |
| Protocol version | 3 | Date and version identifier | 3 |
| Funding | 4 | Sources and types of financial, material, and other support | 21 |
| Roles and responsibilities | 5a | Names, affiliations, and roles of protocol contributors | 21 |
| | 5b | Name and contact information for the trial sponsor | 21 |
| | 5c | Role of study sponsor and funders, if any, in study design; collection, management, analysis, and interpretation of data; writing of the report; and the decision to submit the report for publication, including whether they will have ultimate authority over any of these activities | 21 |
| | 5d | Composition, roles, and responsibilities of the coordinating centre, steering committee, endpoint adjudication committee, data management team, and other individuals or groups overseeing the trial, if applicable (see Item 21a for data monitoring committee) | 7 |
| **Introduction** | | | |
| Background and rationale | 6a | Description of research question and justification for undertaking the trial, including summary of relevant studies (published and unpublished) examining benefits and harms for each intervention | 3 |
| | 6b | Explanation for choice of comparators | 10 |
| Objectives | 7 | Specific objectives or hypotheses | 6 |

For peer review only

| | | | |
|---|---|---|---|
| Trial design | 8 | Description of trial design including type of trial (eg, parallel group, crossover, factorial, single group), allocation ratio, and framework (eg, superiority, equivalence, noninferiority, exploratory) | 6 |
| **Methods: Participants, interventions, and outcomes** | | | |
| Study setting | 9 | Description of study settings (eg, community clinic, academic hospital) and list of countries where data will be collected. Reference to where list of study sites can be obtained | 6 |
| Eligibility criteria | 10 | Inclusion and exclusion criteria for participants. If applicable, eligibility criteria for study centres and individuals who will perform the interventions (eg, surgeons, psychotherapists) | 8,9 |
| Interventions | 11a | Interventions for each group with sufficient detail to allow replication, including how and when they will be administered | 7 |
| | 11b | Criteria for discontinuing or modifying allocated interventions for a given trial participant (eg, drug dose change in response to harms, participant request, or improving/worsening disease) | 9 |
| | 11c | Strategies to improve adherence to intervention protocols, and any procedures for monitoring adherence (eg, drug tablet return, laboratory tests) | 11 |
| | 11d | Relevant concomitant care and interventions that are permitted or prohibited during the trial | 17 |
| Outcomes | 12 | Primary, secondary, and other outcomes, including the specific measurement variable (eg, systolic blood pressure), analysis metric (eg, change from baseline, final value, time to event), method of aggregation (eg, median, proportion), and time point for each outcome. Explanation of the clinical relevance of chosen efficacy and harm outcomes is strongly recommended | 9 |
| Participant timeline | 13 | Time schedule of enrolment, interventions (including any run-ins and washouts), assessments, and visits for participants. A schematic diagram is highly recommended (see Figure) | 11 |
| Sample size | 14 | Estimated number of participants needed to achieve study objectives and how it was determined, including clinical and statistical assumptions supporting any sample size calculations | 17 |
| Recruitment | 15 | Strategies for achieving adequate participant enrolment to reach target sample size | 9 |
| **Methods: Assignment of interventions (for controlled trials)** | | | |
| Allocation: | | | 10 |

| | | | |
|---|---|---|---|
| Sequence generation | 16a | Method of generating the allocation sequence (eg, computer-generated random numbers), and list of any factors for stratification. To reduce predictability of a random sequence, details of any planned restriction (eg, blocking) should be provided in a separate document that is unavailable to those who enrol participants or assign interventions | 10 |
| Allocation concealment mechanism | 16b | Mechanism of implementing the allocation sequence (eg, central telephone; sequentially numbered, opaque, sealed envelopes), describing any steps to conceal the sequence until interventions are assigned | 10 |
| Implementation | 16c | Who will generate the allocation sequence, who will enrol participants, and who will assign participants to interventions | 10 |
| Blinding (masking) | 17a | Who will be blinded after assignment to interventions (eg, trial participants, care providers, outcome assessors, data analysts), and how | 10 |
| | 17b | If blinded, circumstances under which unblinding is permissible, and procedure for revealing a participant's allocated intervention during the trial | 10 |
| **Methods: Data collection, management, and analysis** | | | |
| Data collection methods | 18a | Plans for assessment and collection of outcome, baseline, and other trial data, including any related processes to promote data quality (eg, duplicate measurements, training of assessors) and a description of study instruments (eg, questionnaires, laboratory tests) along with their reliability and validity, if known. Reference to where data collection forms can be found, if not in the protocol | 11 |
| | 18b | Plans to promote participant retention and complete follow-up, including list of any outcome data to be collected for participants who discontinue or deviate from intervention protocols | 11 |
| Data management | 19 | Plans for data entry, coding, security, and storage, including any related processes to promote data quality (eg, double data entry; range checks for data values). Reference to where details of data management procedures can be found, if not in the protocol | 18 |
| Statistical methods | 20a | Statistical methods for analysing primary and secondary outcomes. Reference to where other details of the statistical analysis plan can be found, if not in the protocol | 18 |
| | 20b | Methods for any additional analyses (eg, subgroup and adjusted analyses) | 18 |
| | 20c | Definition of analysis population relating to protocol non-adherence (eg, as randomised analysis), and any statistical methods to handle missing data (eg, multiple imputation) | 18 |

**Methods: Monitoring**

| | | | |
|---|---|---|---|
| Data monitoring | 21a | Composition of data monitoring committee (DMC); summary of its role and reporting structure; statement of whether it is independent from the sponsor and competing interests; and reference to where further details about its charter can be found, if not in the protocol. Alternatively, an explanation of why a DMC is not needed | 17 |
| | 21b | Description of any interim analyses and stopping guidelines, including who will have access to these interim results and make the final decision to terminate the trial | 17 |
| Harms | 22 | Plans for collecting, assessing, reporting, and managing solicited and spontaneously reported adverse events and other unintended effects of trial interventions or trial conduct | 9,17 |
| Auditing | 23 | Frequency and procedures for auditing trial conduct, if any, and whether the process will be independent from investigators and the sponsor | 7 |

**Ethics and dissemination**

| | | | |
|---|---|---|---|
| Research ethics approval | 24 | Plans for seeking research ethics committee/institutional review board (REC/IRB) approval | 3 |
| Protocol amendments | 25 | Plans for communicating important protocol modifications (eg, changes to eligibility criteria, outcomes, analyses) to relevant parties (eg, investigators, REC/IRBs, trial participants, trial registries, journals, regulators) | 3 |
| Consent or assent | 26a | Who will obtain informed consent or assent from potential trial participants or authorised surrogates, and how (see Item 32) | 9 |
| | 26b | Additional consent provisions for collection and use of participant data and biological specimens in ancillary studies, if applicable | 9 |
| Confidentialit y | 27 | How personal information about potential and enrolled participants will be collected, shared, and maintained in order to protect confidentiality before, during, and after the trial | 3 |
| Declaration of interests | 28 | Financial and other competing interests for principal investigators for the overall trial and each study site | 22 |
| Access to data | 29 | Statement of who will have access to the final trial dataset, and disclosure of contractual agreements that limit such access for investigators | 19 |
| Ancillary and post-trial care | 30 | Provisions, if any, for ancillary and post-trial care, and for compensation to those who suffer harm from trial participation | 10 |

For peer review only

| | | | |
|---|---|---|---|
| Dissemination policy | 31a | Plans for investigators and sponsor to communicate trial results to participants, healthcare professionals, the public, and other relevant groups (eg, via publication, reporting in results databases, or other data sharing arrangements), including any publication restrictions | 3 |
| | 31b | Authorship eligibility guidelines and any intended use of professional writers | 21 |
| | 31c | Plans, if any, for granting public access to the full protocol, participant-level dataset, and statistical code | 3 |
| **Appendices** | | | |
| Informed consent materials | 32 | Model consent form and other related documentation given to participants and authorised surrogates | 3 |
| Biological specimens | 33 | Plans for collection, laboratory evaluation, and storage of biological specimens for genetic or molecular analysis in the current trial and for future use in ancillary studies, if applicable | 13 |

*It is strongly recommended that this checklist be read in conjunction with the SPIRIT 2013 Explanation & Elaboration for important clarification on the items. Amendments to the protocol should be tracked and dated. The SPIRIT checklist is copyrighted by the SPIRIT Group under the Creative Commons "Attribution-NonCommercial-NoDerivs 3.0 Unported" license.

For peer review only

# CONSORT 2010 checklist of information to include when reporting a randomised trial*

| Section/Topic | Item No | Checklist item | Reported on page No |
|---|---|---|---|
| **Title and abstract** | | | |
| | 1a | Identification as a randomised trial in the title | 1 |
| | 1b | Structured summary of trial design, methods, results, and conclusions (for specific guidance see CONSORT for abstracts) | 3-4 |
| **Introduction** | | | |
| Background and objectives | 2a | Scientific background and explanation of rationale | 5 |
| | 2b | Specific objectives or hypotheses | 5 |
| **Methods** | | | |
| Trial design | 3a | Description of trial design (such as parallel, factorial) including allocation ratio | 6 |
| | 3b | Important changes to methods after trial commencement (such as eligibility criteria), with reasons | 8 |
| Participants | 4a | Eligibility criteria for participants | 8 |
| | 4b | Settings and locations where the data were collected | 7 |
| Interventions | 5 | The interventions for each group with sufficient details to allow replication, including how and when they were actually administered | 9 |
| Outcomes | 6a | Completely defined pre-specified primary and secondary outcome measures, including how and when they were assessed | 9 |
| | 6b | Any changes to trial outcomes after the trial commenced, with reasons | 9 |
| Sample size | 7a | How sample size was determined | 17 |
| | 7b | When applicable, explanation of any interim analyses and stopping guidelines | 17 |
| Randomisation: | | | |
| Sequence generation | 8a | Method used to generate the random allocation sequence | 10 |
| | 8b | Type of randomisation; details of any restriction (such as blocking and block size) | 10 |
| Allocation concealment mechanism | 9 | Mechanism used to implement the random allocation sequence (such as sequentially numbered containers), describing any steps taken to conceal the sequence until interventions were assigned | 10 |
| Implementation | 10 | Who generated the random allocation sequence, who enrolled participants, and who assigned participants to interventions | 10 |
| Blinding | 11a | If done, who was blinded after assignment to interventions (for example, participants, care providers, those | 9,10 |



For peer review only

For peer review only - http://bmjopen.bmj.com/site/about/guidelines.xhtml

| | | | |
|---|---|---|---|
| | | assessing outcomes) and how | |
| | 11b | If relevant, description of the similarity of interventions | 18 |
| Statistical methods | 12a | Statistical methods used to compare groups for primary and secondary outcomes | 18 |
| | 12b | Methods for additional analyses, such as subgroup analyses and adjusted analyses | 18 |
| **Results** | | | |
| Participant flow (a diagram is strongly recommended) | 13a | For each group, the numbers of participants who were randomly assigned, received intended treatment, and were analysed for the primary outcome | 9 |
| | 13b | For each group, losses and exclusions after randomisation, together with reasons | 17 |
| Recruitment | 14a | Dates defining the periods of recruitment and follow-up | 13 |
| | 14b | Why the trial ended or was stopped | 9 |
| Baseline data | 15 | A table showing baseline demographic and clinical characteristics for each group | 11,12,13 |
| Numbers analysed | 16 | For each group, number of participants (denominator) included in each analysis and whether the analysis was by original assigned groups | 18 |
| Outcomes and estimation | 17a | For each primary and secondary outcome, results for each group, and the estimated effect size and its precision (such as 95% confidence interval) | 18 |
| | 17b | For binary outcomes, presentation of both absolute and relative effect sizes is recommended | 18 |
| Ancillary analyses | 18 | Results of any other analyses performed, including subgroup analyses and adjusted analyses, distinguishing pre-specified from exploratory | 18 |
| Harms | 19 | All important harms or unintended effects in each group (for specific guidance see CONSORT for harms) | 9,17 |
| **Discussion** | | | |
| Limitations | 20 | Trial limitations, addressing sources of potential bias, imprecision, and, if relevant, multiplicity of analyses | 21 |
| Generalisability | 21 | Generalisability (external validity, applicability) of the trial findings | 21 |
| Interpretation | 22 | Interpretation consistent with results, balancing benefits and harms, and considering other relevant evidence | 21 |
| **Other information** | | | |
| Registration | 23 | Registration number and name of trial registry | 3 |
| Protocol | 24 | Where the full trial protocol can be accessed, if available | 3 |
| Funding | 25 | Sources of funding and other support (such as supply of drugs), role of funders | 21 |

*We strongly recommend reading this statement in conjunction with the CONSORT 2010 Explanation and Elaboration for important clarifications on all the items. If relevant, we also recommend reading CONSORT extensions for cluster randomised trials, non-inferiority and equivalence trials, non-pharmacological treatments, herbal interventions, and pragmatic trials. Additional extensions are forthcoming: for those and for up to date references relevant to this checklist, see www.consort-statement.org.



For peer review only - http://bmjopen.bmj.com/site/about/guidelines.xhtml

# Effects of Continuous Positive Airway Pressure on Neurocognitive Architecture and Function in Patients with Obstructive Sleep Apnea: Study Protocol for a Multicenter Randomized Controlled Trial


| | |
|---|---|
| Journal: | *BMJ Open* |
| Manuscript ID | bmjopen-2016-014932.R2 |
| Article Type: | Protocol |
| Date Submitted by the Author: | 04-Feb-2017 |



| | |
|---|---|
| Complete List of Authors: | Xu, Huajun; Shanghai 6th Peoples Hospital Affiliated to Shanghai Jiaotong University School of Medicine, Department of Otolaryngology Head and Neck Surgery & Center of Sleep Medicine<br>Wang, Hui; Shanghai 6th Peoples Hospital Affiliated to Shanghai Jiaotong University School of Medicine, Department of Otolaryngology Head and Neck Surgery & Center of Sleep Medicine<br>Guan, Jian; Shanghai 6th Peoples Hospital Affiliated to Shanghai Jiaotong University School of Medicine, Department of Otolaryngology Head and Neck Surgery & Center of Sleep Medicine<br>Yi, Hongliang; Shanghai 6th Peoples Hospital Affiliated to Shanghai Jiaotong University School of Medicine, Department of Otolaryngology Head and Neck Surgery & Center of Sleep Medicine<br>Qian, Yingjun; Shanghai 6th Peoples Hospital Affiliated to Shanghai Jiaotong University School of Medicine, Department of Otolaryngology Head and Neck Surgery & Center of Sleep Medicine<br>Zou, Jianyin; Shanghai 6th Peoples Hospital Affiliated to Shanghai Jiaotong University School of Medicine, Department of Otolaryngology Head and Neck Surgery & Center of Sleep Medicine<br>Xia, Yunyan; Shanghai 6th Peoples Hospital Affiliated to Shanghai Jiaotong University School of Medicine, Department of Otolaryngology Head and Neck Surgery & Center of Sleep Medicine<br>Fu, Yiqun; Shanghai 6th Peoples Hospital Affiliated to Shanghai Jiaotong University School of Medicine, Department of Otolaryngology Head and Neck Surgery & Center of Sleep Medicine<br>Li, Xinyi; Shanghai 6th Peoples Hospital Affiliated to Shanghai Jiaotong University School of Medicine, Department of Otolaryngology Head and Neck Surgery & Center of Sleep Medicine<br>Jiao, Xiao; Shanghai 6th Peoples Hospital Affiliated to Shanghai Jiaotong University School of Medicine, Department of Otolaryngology Head and Neck Surgery & Center of Sleep Medicine<br>Huang, Hengye; Shanghai Jiao Tong University, Department of Epidemiology, School of Public Health<br>Dong, Pin; Shanghai Jiaotong University First People's Hospital, Department of Otorhinolaryngology<br>YU, Ziwei; Shanghai Jiaotong University First People's Hospital, Department of Otorhinolaryngology<br>Yang, Jun; Shanghai Jiaotong University School of Medicine Xinhua Hospital, Department of Otolaryngology Head and Neck Surgery<br>Xiang, Mingliang; Shanghai Jiaotong University School of Medicine Xinhua |

| | |
|---|---|
| | Hospital, Department of Otolaryngology Head and Neck Surgery<br>Li, Jiping; Shanghai Jiao Tong University School of Medicine Affiliated Renji Hospital, Department of Otolaryngology Head and Neck Surgery<br>Chen, Yanqing; Shanghai Jiao Tong University School of Medicine Affiliated Renji Hospital, Department of Otolaryngology Head and Neck Surgery<br>Wang, Peihua; Shanghai 9th Peoples Hospital Affiliated to Shanghai Jiaotong University School of Medicine, Department of Otolaryngology Head and Neck Surgery<br>Sun, Yizhou; Shanghai 9th Peoples Hospital Affiliated to Shanghai Jiaotong University School of Medicine, Department of Otolaryngology Head and Neck Surgery<br>Li, Yuehua; Shanghai 6th Peoples Hospital Affiliated to Shanghai Jiaotong University School of Medicine, Department of Radiology<br>Zheng, Xiaojian; Shanghai 6th Peoples Hospital Affiliated to Shanghai Jiaotong University School of Medicine, Shanghai Key Laboratory of Diabetes Mellitus and Center for Translational Medicine<br>Jia, Wei; Shanghai 6th Peoples Hospital Affiliated to Shanghai Jiaotong University School of Medicine, Shanghai Key Laboratory of Diabetes Mellitus and Center for Translational Medicine<br>Yin, Shankai; Shanghai 6th Peoples Hospital Affiliated to Shanghai Jiaotong University School of Medicine, Department of Otolaryngology Head and Neck Surgery & Center of Sleep Medicine |
| <b>Primary Subject Heading</b>: | Ear, nose and throat/otolaryngology |
| Secondary Subject Heading: | Ear, nose and throat/otolaryngology, Neurology |
| Keywords: | obstructive sleep apnea, continuous positive airway pressure, cognitive function, gut microbiota, metabolomics |
| | |

SCHOLARONE™
Manuscripts

review only

# Effects of Continuous Positive Airway Pressure on Neurocognitive Architecture and Function in Patients with Obstructive Sleep Apnea: Study Protocol for a Multicenter Randomized Controlled Trial

**Running title**: Effects of CPAP on Neurocognitive Architecture and Function in Patients with OSA

Xu H MD,Ph D[1,2,3*], Wang H MD,Ph D [1,2,3*], Guan J MD,Ph D [1,2,3], Yi H MD, Ph D [1,2,3], Qian Y MD,Ph D [1,2,3], Zou J MD,Ph D [1,2,3], Xia Y MD [1,2,3], Fu Y MD[1,2,3], Li X MD[1,2,3], Jiao X MD[1,2,3], Huang H MD [4], Dong P MD,Ph D [5], Yu Z, MD,Ph D [5], Yang J MD,Ph D[6], Xiang M MD,Ph D[6], Li J MD,Ph D [7], Chen Y MD,Ph D [7],Wang P MD,Ph D [8], Sun Y, MD,Ph D [8], Li Y MD,Ph D[9], Zheng X,Ph D [10], Jia W MD,Ph D [10,11] , Yin S MD,Ph D [1,2,3]

Both Xu H and Wang H contributed equally to this paper

Corresponding to Guan J & Yin S, guanjian0606@sina.com, skyin@sjtu.edu.cn

1 Department of Otolaryngology Head and Neck Surgery & Center of Sleep Medicine, Shanghai Jiao Tong University Affiliated Sixth People's Hospital, 600 Yishan Road, 200233 Shanghai, China.

2 Otolaryngological Institute of Shanghai Jiao Tong University, 600 Yishan Road, 200233 Shanghai, China.



For peer review only - http://bmjopen.bmj.com/site/about/guidelines.xhtml

3 Clinical Research Center, Shanghai Jiao Tong University School of Medicine, 225 South Chongqing Road, 200020 Shanghai, China.

4 Department of Epidemiology, School of Public Health, Shanghai Jiao Tong University, 225South Chongqing Road, 200020 Shanghai, China.

5Department of Otorhinolaryngology, Shanghai First People's Hospital, Shanghai Jiao Tong University, 100 Haining Road, 200080 Shanghai, China.

6 Department of Otolaryngology Head and Neck Surgery, Xinhua Hospital, Affiliated to Shanghai Jiaotong University School of Medicine, 1665 Kongjiang Road, 200092 Shanghai, China.

7 Department of Otorhinolaryngology Head and Neck Surgery, Ren Ji Hospital, School of Medicine, Shanghai Jiao Tong University, 160 Pu Jian Road, Shanghai, 200127, China.

8 Department of Otolaryngology Head and Neck Surgery, Shanghai Ninth People's Hospital, Affiliated to Shanghai Jiaotong University School of Medicine, 500 Quxi Road, Shanghai, 200000, China.

9 Department of Radiology, Shanghai Jiao Tong University Affiliated Sixth People's Hospital, 600 Yi Shan Road, Shanghai 200233, China.

10 Shanghai Key Laboratory of Diabetes Mellitus and Center for Translational Medicine, Shanghai Jiao Tong University Affiliated Sixth People's Hospital, , 600 Yishan Road, Shanghai, 200233, China.

11 University of Hawaii Cancer Center, Honolulu, Hawaii, 96813, USA.



For peer review only - http://bmjopen.bmj.com/site/about/guidelines.xhtml

For peer review only

## Abstract

***Objectives***

Many clinical studies have indicated that obstructive sleep apnea (OSA), the most common chronic sleep disorder, may affect neurocognitive function, and that treatment for continuous positive airway pressure (CPAP) has some neurocognitive protective effects against the adverse effects of OSA. However, the effects of CPAP treatment on neurocognitive architecture and function remain unclear. Therefore, this multicenter trial was designed to investigate whether and when neurocognitive architecture and function in patients with OSA can be improved by CPAP treatment, and to explore the role of gut microbiota in improving neurocognitive function during treatment.

***Methods/Design***

This study will be a multicenter, randomized, controlled trial with allocation concealment and assessor blinding. A total of 148 eligible patients with moderate to severe OSA will be enrolled from five sleep centers and randomized to receive CPAP with best supportive care (BSC) intervention or BSC intervention alone. Cognitive function, structure and function of brain regions, gut microbiota, metabolites, biochemical variables, electrocardiography, echocardiography, pulmonary function and arterial stiffness will be assessed at baseline before randomization and at 3, 6 and 12 months.




For peer review only - http://bmjopen.bmj.com/site/about/guidelines.xhtml

### *Ethics and Dissemination*

Ethics approval was given by the Medical Ethics Committee of Shanghai Jiao Tong University Affiliated Sixth People's Hospital (approval number 2015-79). The findings from this study will be disseminated in peer-reviewed journals and at conferences.

**Trial registration number**: NCT02886156; Pre-results.

**Keywords:** obstructive sleep apnea, continuous positive airway pressure, cognitive function, gut microbiota, metabolomics

**Article summary**

Strengths and limitations of this study:

1) Multicenter, randomized, prospective study.

2) Relatively large number of participants (n=148).

3) Strictly standardized study population.

4) The multicenter pragmatic design strengthens future results and generalizability.

5) Possible selection bias may exist, as not all possible patients may enter in the study.



For peer review only - http://bmjopen.bmj.com/site/about/guidelines.xhtml

**Background**

Obstructive sleep apnea (OSA) is a common chronic sleep disorder characterized by recurrent partial or complete pharyngeal obstruction during sleep.[1] There is a high prevalence (2–4%) of this condition in adults aged 30–60 years, which is increasing with the growing epidemic of obesity.[2-4] OSA is well recognized as a risk factor for neurocognitive, cardiovascular and metabolic disorders.[5-7] It is worth noting that neurocognitive dysfunction is associated with a series of sequelae, such as social and economic burdens.[8-10] Continuous positive airway pressure (CPAP) is typically recommended as first-line treatment for OSA, as it can partly or completely reverse OSA-related complications, including cognitive impairments.[11 12] In addition, CPAP may be effective for reversing structural and functional brain changes.[13 14]

To date, only two randomized controlled trials (RCTs) have simultaneously evaluated the effects of CPAP on cognition and neuroimaging; both studies had relatively small sample sizes and short intervention periods.[15 16] However, the response to CPAP treatment in the trials was variable. Thus, the relationships among OSA, brain and neurobehavioral function are complex and must be comprehensively evaluated, including how, when and how long CPAP treatment should be applied for effective therapy.

The gut microbiota is an important factor that affects neurological functions,[17] and may be involved in the modulation of central nervous function (e.g., the hypothalamus–pituitary–adrenal axis) and associated with neuropsychiatric



For peer review only - http://bmjopen.bmj.com/site/about/guidelines.xhtml

conditions.[17 18] The composition of the gut microbiota is altered through intermittent hypoxia or sleep fragmentation in animal models.[19 20] However, there have been no studies to determine whether changes in human intestinal microbiota are associated with OSA, and whether or how the microbiota can be modulated by CPAP. This study will clarify the complex relationships among OSA, gut microbiota and neurological functions.

## Methods/Design

### *Study aims and hypotheses*

The primary aim of this study is to assess the dynamic (3, 6 and 12 months) effects of CPAP on cognitive function, neurocognitive architecture and function. The primary hypothesis of this study is that, compared with the non-CPAP treatment group, patients with OSA who receive CPAP intervention will show improved cognitive function through structural and functional brain modulation immediately after 3 months of CPAP treatment, and that these benefits will persist at the 6-month and 12-month follow-ups. The secondary aims of this study are to investigate whether and which sleep parameters are correlated with improvements of cognitive function, neurocognitive architecture and function; to explore whether the metabolic status assessed by metabolomics can be reversed by CPAP and correlated with improvement of neurocognitive impairment; and to investigate whether CPAP modulates the gut microbiota and contributes to alleviation of neurocognitive impairment. We hypothesize that patients with OSA who undergo



For peer review only - http://bmjopen.bmj.com/site/about/guidelines.xhtml

CPAP treatment will have metabolic improvement and modulation of gut microbiota, and that these changes will have beneficial effects on neurocognitive impairment.

*Study design*

The Shanghai Multicenter Obstructive Sleep Apnea Therapy trial is a multicenter, evaluator-blinded, randomized, parallel controlled trial performed in five sleep centers (Department of Otolaryngology & Head and Neck Surgery, Shanghai Jiao Tong University Affiliated Sixth People's Hospital; Department of Otolaryngology-Head and Neck Surgery, Shanghai First People's Hospital, Shanghai Jiao Tong University; Department of Otolaryngology, Renji Hospital, School of Medicine, Shanghai Jiao Tong University; Department of Otolaryngology, Head and Neck Surgery, Xinhua Hospital, Shanghai Jiao Tong University School of Medicine; Department of Otolaryngology, The Ninth People's Hospital Affiliated to Shanghai Jiao-Tong University School of Medicine) in the Shanghai district. The design of this protocol strictly followed the Standard Protocol Items Recommendations for Interventional Trials,[21] and the Consolidated Standards of Reporting Trials statements.[22] The study will enroll 148 eligible participants with moderate to severe OSA from the five abovementioned sleep centers and randomize them to receive CPAP with best supportive care (BSC) intervention or BSC intervention alone. Cognitive function, structure and function of brain regions, metabolites, gut microbiota, biochemical variables, electrocardiography, echocardiography, pulmonary function and arterial stiffness will be assessed at baseline



For peer review only - http://bmjopen.bmj.com/site/about/guidelines.xhtml

before randomization and at 3, 6 and 12 months. The flow diagram of this RCT is presented in Figure 1.

***Study population***

The study population (30–65 years old) will be enrolled from Shanghai and the surrounding area, and all of the participants will be from the Han population. The diagnostic, inclusion and exclusion criteria for this trial will be as described below.

***Diagnostic criteria***

According to the American Academic Sleep Medicine (AASM) 2007 criteria,[23] moderate and severe OSA are defined as an apnea-hypopnea index (AHI) ≥ 15 and 30 events per hour, respectively, and the normal state is defined as AHI < 5 events per hour.

***Eligible participants***

*Inclusion criteria*

1) Informed consent
2) Age 30–65 years
3) Newly diagnosed OSA (full-night in-laboratory polysomnography [PSG] with an AHI ≥ 15 events per hour)
4) Ability to tolerate CPAP treatment based on 1 night of use (must use it for at least 4 hours)
5) No participation in any other clinical trial in the past 3 months
6) Able to accomplish relevant tests and follow-up



For peer review only - http://bmjopen.bmj.com/site/about/guidelines.xhtml

*Exclusion criteria*

1) Severe systemic diseases (e.g., cardiac, hepatic and renal failure)
2) Psychiatric conditions (e.g., depression, mania, schizophrenia)
3) Neurological diseases (e.g., head trauma, brain tumor, epilepsy, stroke, transient ischemic attack, coma)
4) Sleep disorders other than OSA (narcolepsy, insomnia, chronic sleep deprivation, rapid eye movement [REM] behavior disorder and restless legs syndrome, central sleep apnea (≥50% of AHI composed of central apneas) or obesity hypoventilation syndrome)
5) Alcoholism (according to the National Institute on Alcohol Abuse and Alcoholism, alcoholism is defined as alcohol consumption exceeding 14 standard drinks per week or four drinks per day in men or exceeding seven standard drinks per week or three drinks per day in women. A standard drink was defined as one 12-ounce bottle of beer, one 5-ounce glass of wine or 1.5 ounces of distilled spirits) [24], drug addiction, use of psychotropic drugs, current sedative use or narcotics
6) Prior therapy for OSA (i.e., CPAP, upper airway surgery, oral appliance)
7) Mini Mental State Examination (MMSE) score < 24
8) Left-handed
9) Magnetic resonance imaging (MRI) contraindications (e.g., claustrophobic or metal implantation)



For peer review only - http://bmjopen.bmj.com/site/about/guidelines.xhtml

10) Gastrointestinal surgery during the last year, except for appendicitis and hernia surgery

11) Pregnancy

12) Use of intestinal flora regulator (e.g., antibiotics or probiotics) in the previous 8 weeks

13) Medical treatment for cholecystitis, gallstones, gastrointestinal ulcers, urinary tract infection, acute pyelonephritis or cystitis in the past 3 months

14) Infectious diseases, such as tuberculosis, acquired immune deficiency syndrome

15) Commercial drivers or people deemed to be at risk of driving-related accidents

16) Deemed by the researchers to be unsuitable for this trial

***Withdrawal criteria and management***

Subjects will be allowed or required to withdraw from this trial if they have insurmountable claustrophobia or other unbearable conditions associated with CPAP use, major protocol violation, or the development of a serious disease that prevents continuation in the trial.

***Recruitment and screening***

Participants will be recruited from Shanghai and its vicinity through newspapers and instant messaging software (WeChat). Potential participants with self-reported severe snoring and with an interest in participating will be screened by a standard PSG and examined for eligibility (AHI ≥ 15 times per hour) for multicenter RCT. Eligible participants will be given information about this trial and have an informed discussion



For peer review only - http://bmjopen.bmj.com/site/about/guidelines.xhtml

with trained research assistants regarding the information provided. Written informed consent will be obtained from each participant prior to enrollment.

***Randomization, allocation concealment and blinding***

After the baseline assessment, eligible patients with moderate to severe OSA will be randomly and equally allocated (randomization ratio 1:1) to the CPAP plus BSC group or BSC only group. The central random allocation sequence will be produced using the statistical software SAS v.9.4 and managed by an independent statistician (from the Department of Epidemiology, School of Public Health, Shanghai Jiao Tong University) blinded to the recruitment, evaluation and intervention of the participants. The research assistant from each sleep center will request (via E-mail) a participant's allocation from the statistician. The group allocation will not be masked by participants or intervention supervisors in this trial; however, the patients' group assignments can be blinded to outcome assessors and data statisticians (i.e., which patients are allocated to the CPAP treatment versus BSC groups will not be disclosed).

***Intervention***

*BSC only group*

Participants in the BSC only group will receive advice regarding lifestyle modification, sleep hygiene, naps, exercise, caffeine, and diet, and avoiding alcohol consumption, but no specific weight loss program, diet or salt restriction will be suggested.



For peer review only - http://bmjopen.bmj.com/site/about/guidelines.xhtml

*CPAP plus BSC group*

Participants in the CPAP plus BSC group will receive CPAP treatment plus the aforementioned BSC intervention. CPAP treatment (LOTUS AUTO; Curative Medical Technology, Beijing, China) will be initiated using standard clinical practice (before CPAP treatment, the standard clinical practice is for the doctors to educate patients on the use of CPAP and provide positive encouragement) at each center. Online monitoring of CPAP compliance can also be used to improve compliance. Participants in both groups will be asked to continue their usual medical care during the trial.

***Follow-up***

At 3, 6 and 12 months during the intervention period, all of the participants will undergo the same examinations as at baseline. During the follow-up period, CPAP usage information will be automatically downloaded daily from auto CPAP via the Internet.

***Outcome assessment***

The variables in this trial consist of basic characteristics, primary outcomes and secondary outcomes. The basic characteristics will be measured at baseline, and primary and secondary outcomes will be measured at baseline and at the end of each intervention period (3, 6 and 12 months after randomization). All of the primary and secondary outcomes will be assessed by experienced staff at each sleep center who will be blinded to treatment allocation.



For peer review only - http://bmjopen.bmj.com/site/about/guidelines.xhtml

***Basic characteristics***

Participants' demographic characteristics (e.g., age, sex, years of education, medical history, medication use, occupation) will be collected by recruiters using a questionnaire. The basic Montreal Cognitive Assessment (MoCA) and MMSE scores will be assessed using the corresponding standardized scales. The assessors in each sleep center will be trained by a neurologist. Baseline assessment will be completed before randomization.

***Primary and secondary outcomes***

*Primary endpoints*

MoCA

*Secondary endpoints*

MMSE

Default mode network and cortical thickness measured by functional and structural MRI.

Cerebral metabolites, such as N-acetylaspartate (NAA), choline (Cho), creatine (Cr), glutamate and glutamine and myo-inositol measured by magnetic resonance spectroscopy (MRS).

Neuropsychological tests will also be performed, including mental arithmetic, memory scanning, movement perception, switching attention, space location memory span and attention allocation choice reaction time curve fit.



For peer review only - http://bmjopen.bmj.com/site/about/guidelines.xhtml

Sleep variables (apnea-hypopnea index [AHI], oxygen desaturation index [ODI], lowest oxygen saturation [LSaO$_2$]) mean oxygen saturation [mean SaO$_2$], micro-arousal index [MAI], total sleep time, total REM sleep time, total slow wave sleep time, etc.).

*Auditory Testing*

Central auditory processing testing (Speech-in-noise test, Competing sentences test, Gap detection, Dichotic listening)

256-channel high-density EEG recordings

The gut microbial community will be quantified using metagenomics analysis.[25]

Low-molecular-weight metabolites (≤1000 kDa) will be quantified by metabolomics analysis.[26]

Amyloid β protein 42, presenilin 1, amyloid-β protein 40, total GSK-3β and serine-9 phosphorylated GSK-3β

*Other outcomes*

*Biochemical variables*: lipid profile (total cholesterol, triglycerides, high-density lipoprotein cholesterol, low-density lipoprotein cholesterol, apoA-I, apoB, apoE, and lipoprotein(a)), glucose, insulin, HbA1c, free fatty acids, cholinesterase, lipopolysaccharide binding protein, toll-like receptor-4, TLR-2, hepatic function tests (alanine aminotransferase, aspartate aminotransferase, γ-glutamyl endopeptidase, alkaline phosphatase, total protein, albumin, total bilirubin, direct bilirubin, bile acid, prealbumin), renal function tests (creatinine, urea nitrogen, uric acid), microalbuminuria; apolipoprotein E genotyping



For peer review only - http://bmjopen.bmj.com/site/about/guidelines.xhtml

*Inflammatory biomarkers*: leptin, RANTES, monocyte chemotactic factor 1, interleukin (IL)-6, IL-8, tumor necrosis factor-α, hypersensitive C-reactive protein, resistin, serum amyloid A protein, α-acid glycoprotein

*Anti-inflammatory biomarkers*: adiponectin, IL-1Ra

*Oxidative stress index*: 8-isoprostane

Blood pressure (systolic blood pressure, diastolic blood pressure)

Anthropometric variables (weight, height, waist, hip, chest and neck circumferences, visceral fat area, subcutaneous fat area)

Echocardiography

Arterial stiffness (pulse wave velocity, ankle brachial index, toe-brachial index)

Lung function (e.g., forced expiratory volume in 1 s, forced vital capacity)

Electrocardiography (ECG)

***Study examination procedures***

All of the study measurements will be performed before and after 3, 6 and 12 months of CPAP intervention at each center, except for lung function and ECG.

*Anthropometric measurements*: Height and weight will be measured using standard methods. BMI (weight [kg]/height$^2$ [m$^2$]) will be calculated from height and weight. Waist, hip and neck circumferences will be measured using a measuring tape in the conventional manner.

*Urine samples*: First midstream urine samples will be collected in the morning with the collected urine samples stored immediately at –80°C for further analyses.



For peer review only - http://bmjopen.bmj.com/site/about/guidelines.xhtml

*Blood samples*: Fasting blood samples will be collected in the morning, and the samples will be immediately sent for further analyses such as biochemical analysis.

*Fecal samples*: Fecal samples will be collected and stored at –80°C until use in subsequent assays. The composition of microbiota will be measured using 16S rRNA-hybridization and DNA staining.

*Sleep respiratory variables*: Laboratory-based PSG (Alice 4 or 5, Respironics; Pittsburgh, PA, USA) will be used to record sleep parameters. Electroencephalography (EEG), electrooculography, ECG, electromyography, nasal/oral airflow, thoracic/abdominal movement, pulse oximetry, body posture and snoring will be recorded. The definition of OSA is established according to the AASM 2007 criteria.

*Neuropsychological tests*: Two questionnaires (MoCA and MMSE) will be administered. Neuropsychological tests will also be performed, including mental arithmetic, memory scanning, movement perception, switching attention, space location memory span and attention allocation choice reaction time curve fit.

*Auditory testing*: Audiograms will be measured in one-octave steps at frequencies ranging from 125 Hz to 8 kHz and then at 10, 12.5, 16 and 20 kHz. DPOAE values will be recorded in both groups using a Madsen Capella cochlear emissions analyzer in a soundproof room. Only patients with normal audiometry (threshold below 25 dB HL at all frequencies from 0.125 to 20 kHz), normal DPOAE levels, normal tympanometric curve of type A, and presence of ipsi- and contralateral stapedial reflexes will be included.



For peer review only

*Central auditory processing testing*: The following central auditory processing tests will be performed in a soundproof room. Before the formal test, participants will be allowed to practice as many times as required and provide feedback to become familiar with the stimuli. (1) Speech-in-noise test: All of the signal processing will be performed with MATLAB software. The speech of 20 ten-word sentences will be presented under both quiet and noisy conditions with speech-shaped noise and will be delivered bilaterally through Sennheiser HD205 headphones at a signal-to-noise ratio of –5 dB. (2) Competing sentences test: Babble noise and the target sentence will be presented bilaterally at an intensity level of 65 dB SL (above speech reception threshold) at a signal-to-noise ratio of –3 dB. (3) Gap detection: The purpose of this evaluation is to determine the smallest interval that the listener can detect. Two stimuli separated in time by a “gap” will be presented and the listener will be asked to detect the gap, essentially discriminating sound from silence. Stimuli as white noise will be bilaterally presented as background noise at intensities 40 dB higher than the hearing threshold. (4) Dichotic listening: Two different numbers will be simultaneously presented to both ears with the subjects instructed to repeat both numbers in any order. The test includes 20 number pairs presented dichotically. The number pairs will be aligned for onset and offset to eliminate any cue for the first number heard. Dichotic listening performance will be scored as the percentage of correctly reported different numbers from the right and left ears in the noise-exposed and control groups.



For peer review only - http://bmjopen.bmj.com/site/about/guidelines.xhtml

*256-Channel high-density EEG recording*: EEG data will be recorded with 256 channels on an EGI HydroCel Geodesic Sensor Net, with Cz used as the reference channel. All of the electrode impedances will be detected and maintained at less than 50 kΩ. The oddball stimuli pattern with speech sound is used in this study (via Eprime 2.0; Psychology Software Tools). The auditory oddball task includes presentation sound of /Ba/, whereas /Da/ is presented as the "oddball" target stimulus. The stimulus /Ba/ is presented in 85% of the trials together with the stimulus /Da/ in 15% of the trials. The whole task consists of a total of 1000 auditory stimuli with random interstimulus intervals ranging from 850 ms to 1450 ms. The sound stimuli with an intensity of 75 dB delivered through two loudspeakers at a distance of 100 cm from the subjects. During the P3 cortical waves test, the subjects will be asked to press a switch held in the right hand each time they detect a target tone with the eyes closed. In addition, they will be asked not to respond to any auditory stimulus while watching silent films during the MMN test. Source analysis of event-related potentials as MMN, describing neural sources of measured scalp potentials, will be estimated with GeoSource (version 2.0).

*Brain imaging*: MRI will be performed using the Siemens 3T Verio scanner (MAGNETOM, Siemens) with a 12-channel coil using the same parameters. High-resolution T1-3D and T2-3D weighted MRI sequences will be conducted for all subjects. *T1-3D axial images*: FOV, 230 mm; TR/TE, 1500/2.96 ms; flip angle, 9°; voxel size, 0.9×0.9×1 $mm^3$; slice thickness, 1 mm; and distance factor, 50%. *T2-3D axial images*: FOV, 230 mm; TR/TE, 1500/2.96 ms; voxel size, 0.9×0.9×1 $mm^3$; slice



For peer review only - http://bmjopen.bmj.com/site/about/guidelines.xhtml

For peer review only

thickness, 1 mm; and distance factor, 50%. In the same position, a s-fMRI BOLD sequence will be acquired in the rest condition, and 240 functional images will be obtained (repetition time 2,000 ms, echo time 30 ms, thickness 4.0 mm, gap 1.2 mm, acquisition matrix 64×64, flip angle 90°, field of view 230 ×230 $mm^2$, 30 axial slices with gradient-recalled echo-planar imaging pulse sequence covering the whole brain). Patients will be asked to keep their eyes closed and not to focus their thoughts on any particular topic or to fall asleep. DKI will be performed in all subjects using 30 gradient directions, six b values and an echo-planar DWI sequence. FOV, 300 mm; TR/TE, 1100/109 ms; matrix size 128×128; and slice thickness 4 mm.

*Cerebral metabolites*: MRS will be performed using the Siemens 3T Verio scanner with a 12-channel coil. A 2.0×2.0×2.0 $cm^3$ voxel of interest will be placed at the frontal lobe. NAA, Cho and Cr will be acquired by a MEGA-PRESS sequence using the following parameters: TE/TR=135/5000 ms; average number 80 (scan time 8 min).

*Abdominal and liver fat measurements*: The hepatic, subcutaneous and visceral fat will be measured by axial scanning of the upper abdomen (from the diaphragm to the lower edge of the liver). The participants will be instructed to hold their breath to reduce the artifacts induced by respiratory movement. Anatomical T1-weighted spin-echo MRI will be obtained using the following parameters: slice thickness 5 mm; slice spacing 2.5 mm.



For peer review only - http://bmjopen.bmj.com/site/about/guidelines.xhtml

***Safety measurements***

Any unexpected adverse events during the intervention period will be monitored, reported to a research assistant, and causality in relation to CPAP intervention and the severity of adverse events will be analyzed. The data we submit will be monitored by the Ethics Committee of Shanghai Jiao Tong University Affiliated Sixth People's Hospital. Serious adverse events will be immediately reported to the ethics committee.

***Sample size calculation***

A previous study indicated that the mean and standard deviation (SD) of MoCA score are 26.1 (2.0) and 27.4 (2.3) before and after CPAP treatment, respectively.[27] With a significance level of 0.05 and statistical power of 0.9, the calculated sample size is 59 for each group. Considering a 20% loss to follow-up, a total of 148 participants will be required for randomization.

***Statistical methods***

Study analysis will involve intention-to-treat (ITT). All of the statistical analyses will be performed using SAS ver. 9.4 (SAS Institute, Cary, NC, USA), with a two-sided *p*-value < 0.05 indicating statistical significance. The Kolmogorov–Smirnov test will be performed to test normality of the distribution. Continuous variables will be expressed as means and SD or median (first third quartile) for continuous variables and percentages for categorical variables. To assess primary and secondary outcomes, the mixed-effects linear model with restricted maximum likelihood will be used to analyze the effect of CPAP treatment on the continuous variables, and logistic regression models



For peer review only - http://bmjopen.bmj.com/site/about/guidelines.xhtml

will be used for dependent categorical variables. In each model, the independent variables are group (CPAP plus BSC group, BSC group), measurement time (e.g., baseline and 3, 6 and 12-month follow-ups), confounding factors (e.g., age, BMI), and any inequality factors between the groups at the baseline assessment. A *post hoc* comparison between groups at different time points will be performed using multiple comparisons. A multiple imputation method will be used to fill in missing data.

***Data collection and management***

All of the data collectors and evaluators will be blinded to the group assignments. The data in this trial will be collected using print-based case report forms, which will be transcribed by a research assistant into web-based case report forms (w-CRFs) with an electronic data capture system (EDC). The EDC will be provided by the School of Public Health of Shanghai Jiao Tong University (Shanghai, China) and will meet the available standards of security. Data in w-CRFs will be stored in the EDC system at separate password-protected locations. The research assistants will be responsible for ensuring the integrity and accuracy of all data when data are transcribed into w-CRFs by checks on value ranges and logical checks. Development and validation of the study database, quality control and extraction of data will be performed according to the procedures of the medical research platform.

***Ethical issues***

This protocol will be conducted in accordance with the Declaration of Helsinki. Ethics approval has been obtained from Shanghai Jiao Tong University Affiliated Sixth



For peer review only - http://bmjopen.bmj.com/site/about/guidelines.xhtml

People's Hospital (Ethics number: 2015–79). Written informed consent will be obtained from each participant with a full discussion about the potential benefits and risks before participation.

***Dissemination***

This study will be published in scientific journals and presented at national conferences in the field of neurology and sleep medicine. Study results will also be sent to study participants and disseminated to researchers, healthcare providers, healthcare professionals, and the general public regardless of the magnitude or direction of effects.

**Discussion**

Increasing numbers of clinical studies have shown that CPAP is an effective therapeutic method for neurocognitive dysfunction in OSA. However, the effects and duration of CPAP treatment on neurocognitive function have not been clearly established. This study focuses on the effects of CPAP on middle-aged patients with moderate to severe OSA. Through a 3-, 6- and 12-month intervention with CPAP treatment, the results from a range of clinical and biochemical assessments will provide clearer information on the effects of treatment in OSA.

Measurements of neurocognitive function before and after intervention in this trial may strengthen evidence for links between OSA and neurocognitive dysfunction, or reveal new associations. Comparing patients with OSA to healthy controls will allow us to identify differences in neurocognitive function between the two groups. In addition, through CPAP intervention, it will be possible to explore the mechanism of



For peer review only - http://bmjopen.bmj.com/site/about/guidelines.xhtml

action of CPAP on neurocognitive function (including structural and functional effects), allowing us to investigate the causal relationships between OSA and neurocognitive dysfunction.

The microbial community structure in rodents was altered when the rodents were subjected to intermittent hypoxia or sleep fragmentation.[19 20 28] In addition, sleep fragmentation-induced metabolic alterations may be mediated by concurrent changes in the gut microbiota.[28] Consequently, gut microbiome-targeted therapeutics may help reduce the major end-organ morbidities of OSA. Through metagenomics and metabolomics approaches, we will determine whether gut microbiota play a pivotal role in OSA-related complications, especially in alleviating neurocognitive dysfunction.

To our knowledge, the proposed study will be the first to investigate the effects of CPAP on neurocognitive dysfunction in OSA and to explore the underlying mechanisms using combined "-omics" approaches. The results of this multicenter trial may be helpful for improving the neurocognitive function of patients with OSA.

This protocol describes a multicenter RCT to assess the effects of CPAP treatment on neurocognitive function in patients with moderate to severe OSA. To reduce potential bias, multiple rigorous methods, such as randomization, blinding of raters and statistical analyzers, and ITT principles, will be implemented. Potential limitations of this protocol should also be addressed. First, sham CPAP is not used as a placebo; therefore neither participants nor trial researchers will be blinded to the protocol. Thus, performance bias may be inevitable. Second, although all of the raters



For peer review only - http://bmjopen.bmj.com/site/about/guidelines.xhtml

have been trained by the same neurologist, there may be heterogeneity in neuropsychological tests in each center.

In summary, this will be the first multicenter RCT to dynamically observe the effects of CPAP on neurocognitive function and to explore the role of the gut microbiota in neurocognitive dysfunction in OSA. The results of this study will help to clarify potential mechanisms for alleviation of OSA and improvement of neurocognitive dysfunction.

**Trial status:** At the time of manuscript submission, the enrollment of volunteers is ongoing.

**Acknowledgements** This study was supported by grants-in-aid from multi-center clinical research project from school of medicine, Shanghai Jiao Tong University (DLY201502). The study sponsor played no role in the design, methods, data management or analysis or in the decision to publish. The corresponding authors have full access to all the data in the study and have final responsibility for the decision to submit for publication.

**Contributors** Xu H, Wang H and Yin S conceived the study, designed the study protocol, and drafted the manuscript. Xu H, Wang H, Guan J, Yi H, Qian Y, Zou J, Xia Y, Fu Y, Li X, Jiao X, Dong P, Yu Z, Yang J, Xiang M, Li J, Chen Y, Wang P, Sun Y, Li Y, Jia W and Yin S participated in the coordination and implementation of the study. Xu H, Wang H and Yin S revised and finalized the study protocol. Yin S is in charge of coordination and direct implementation. Huang H helped to develop the study measures



For peer review only - http://bmjopen.bmj.com/site/about/guidelines.xhtml

and analyses. All authors contributed to drafting the manuscript and have read and approved the final manuscript.

**Competing interests** The authors declare that they have no competing interests.

**Patient consent**: Parents will be informed about the project, the purpose of this study, the right to withdraw. The confidentiality of the data will be explained by project staff, and informed consent will be obtained before inclusion. Confidentiality will be guaranteed, and participation is voluntary. The participants will be informed that the results of the interviews and field study will be included in publications.

**Ethics approval:** This study has received human ethics approval from Medical Ethics Committee of Shanghai Jiao Tong University Affiliated Sixth People's Hospital (approval number 2015-79). The protocol is in full compliance with the standards set forth by the abovementioned Medical Ethics Committee. This protocol includes plans for communicating important protocol modifications, obtaining consent, and confidentiality.

**Protocol amendments:** For communicating important protocol modifications, we will first notify the Medical Ethics Committee of Shanghai Jiao Tong University Affiliated Sixth People's Hospital in accordance with Chinese legislation. After their approval, all the Ethics Committee members and investigators of the participating sites will be informed.

**References:**



For peer review only - http://bmjopen.bmj.com/site/about/guidelines.xhtml

1. Strollo PJ, Jr., Rogers RM. Obstructive sleep apnea. *The New England journal of medicine* 1996;334(2):99-104. doi: 10.1056/nejm199601113340207 [published Online First: 1996/01/11]
2. Young T, Palta M, Dempsey J, et al. The occurrence of sleep-disordered breathing among middle-aged adults. *The New England journal of medicine* 1993;328(17):1230-5. doi: 10.1056/nejm199304293281704 [published Online First: 1993/04/29]
3. Peppard PE, Young T, Barnet JH, et al. Increased prevalence of sleep-disordered breathing in adults. *Am J Epidemiol* 2013;177(9):1006-14. doi: 10.1093/aje/kws342 [published Online First: 2013/04/17]
4. Heinzer R, Vat S, Marques-Vidal P, et al. Prevalence of sleep-disordered breathing in the general population: the HypnoLaus study. *The Lancet Respiratory medicine* 2015;3(4):310-8. doi: 10.1016/s2213-2600(15)00043-0 [published Online First: 2015/02/16]
5. Sanchez-de-la-Torre M, Campos-Rodriguez F, Barbe F. Obstructive sleep apnoea and cardiovascular disease. *The Lancet Respiratory medicine* 2013;1(1):61-72. doi: 10.1016/s2213-2600(12)70051-6 [published Online First: 2013/12/11]
6. Aurora RN, Punjabi NM. Obstructive sleep apnoea and type 2 diabetes mellitus: a bidirectional association. *The Lancet Respiratory medicine* 2013;1(4):329-38. doi: 10.1016/s2213-2600(13)70039-0 [published Online First: 2014/01/17]
7. Rosenzweig I, Glasser M, Polsek D, et al. Sleep apnoea and the brain: a complex relationship. *The Lancet Respiratory medicine* 2015;3(5):404-14. doi: 10.1016/s2213-2600(15)00090-9 [published Online First: 2015/04/19]
8. Sales LV, Bruin VM, D'Almeida V, et al. Cognition and biomarkers of oxidative stress in obstructive sleep apnea. *Clinics (Sao Paulo, Brazil)* 2013;68(4):449-55. doi: 10.6061/clinics/2013(04)03 [published Online First: 2013/06/20]
9. Quera Salva MA, Barbot F, Hartley S, et al. Sleep disorders, sleepiness, and near-miss accidents among long-distance highway drivers in the summertime. *Sleep medicine* 2014;15(1):23-6. doi: 10.1016/j.sleep.2013.06.018 [published Online First: 2013/11/30]
10. Stevenson MR, Elkington J, Sharwood L, et al. The role of sleepiness, sleep disorders, and the work environment on heavy-vehicle crashes in 2 Australian states. *Am J Epidemiol* 2014;179(5):594-601. doi: 10.1093/aje/kwt305 [published Online First: 2013/12/20]
11. Zhou J, Camacho M, Tang X, et al. A review of neurocognitive function and obstructive sleep apnea with or without daytime sleepiness. *Sleep medicine* 2016;23:99-108. doi: 10.1016/j.sleep.2016.02.008 [published Online First: 2016/06/12]
12. Crawford-Achour E, Dauphinot V, Martin MS, et al. Protective Effect of Long-Term CPAP Therapy on Cognitive Performance in Elderly Patients with Severe OSA: The PROOF Study. *Journal of clinical sleep medicine : JCSM : official publication of the American Academy of Sleep Medicine* 2015;11(5):519-24. doi: 10.5664/jcsm.4694 [published Online First: 2015/02/24]
13. Tahmasian M, Rosenzweig I, Eickhoff SB, et al. Structural and functional neural adaptations in obstructive sleep apnea: An activation likelihood estimation meta-analysis. *Neuroscience and biobehavioral reviews* 2016;65:142-56. doi: 10.1016/j.neubiorev.2016.03.026 [published Online First: 2016/04/04]
14. Henderson LA, Fatouleh RH, Lundblad LC, et al. Effects of 12 Months Continuous Positive Airway Pressure on Sympathetic Activity Related Brainstem Function and Structure in Obstructive Sleep



For peer review only

For peer review only - http://bmjopen.bmj.com/site/about/guidelines.xhtml

Apnea. *Frontiers in neuroscience* 2016;10:90. doi: 10.3389/fnins.2016.00090 [published Online First: 2016/03/26]

15. Dalmases M, Sole-Padulles C, Torres M, et al. Effect of CPAP on Cognition, Brain Function, and Structure Among Elderly Patients With OSA: A Randomized Pilot Study. *Chest* 2015;148(5):1214-23. doi: 10.1378/chest.15-0171 [published Online First: 2015/06/13]
16. Rosenzweig I, Glasser M, Crum WR, et al. Changes in Neurocognitive Architecture in Patients with Obstructive Sleep Apnea Treated with Continuous Positive Airway Pressure. *EBioMedicine* 2016;7:221-9. doi: 10.1016/j.ebiom.2016.03.020 [published Online First: 2016/06/21]
17. Rogers GB, Keating DJ, Young RL, et al. From gut dysbiosis to altered brain function and mental illness: mechanisms and pathways. *Molecular psychiatry* 2016;21(6):738-48. doi: 10.1038/mp.2016.50 [published Online First: 2016/04/20]
18. Forsythe P, Sudo N, Dinan T, et al. Mood and gut feelings. *Brain, behavior, and immunity* 2010;24(1):9-16. doi: 10.1016/j.bbi.2009.05.058 [published Online First: 2009/06/02]
19. Moreno-Indias I, Torres M, Montserrat JM, et al. Intermittent hypoxia alters gut microbiota diversity in a mouse model of sleep apnoea. *The European respiratory journal* 2015;45(4):1055-65. doi: 10.1183/09031936.00184314 [published Online First: 2014/12/30]
20. Durgan DJ, Ganesh BP, Cope JL, et al. Role of the Gut Microbiome in Obstructive Sleep Apnea-Induced Hypertension. *Hypertension* 2016;67(2):469-74. doi: 10.1161/hypertensionaha.115.06672 [published Online First: 2015/12/30]
21. Chan AW, Tetzlaff JM, Altman DG, et al. SPIRIT 2013 statement: defining standard protocol items for clinical trials. *Annals of internal medicine* 2013;158(3):200-7. doi: 10.7326/0003-4819-158-3-201302050-00583 [published Online First: 2013/01/09]
22. Schulz KF, Altman DG, Moher D. CONSORT 2010 statement: updated guidelines for reporting parallel group randomized trials. *Annals of internal medicine* 2010;152(11):726-32. doi: 10.7326/0003-4819-152-11-201006010-00232 [published Online First: 2010/03/26]
23. Iber C A-IS, Chesson AL, Jr., Quan SF. for the American Academy of Sleep Medicine. The AASM manual for the scoring of sleep and associated events: rules, terminology and technical specifications. 1st ed. Westchester, IL: American Academy of Sleep Medicine. 2007
24. Guan J, Yi H, Zou J, et al. Distinct severity stages of obstructive sleep apnoea are correlated with unique dyslipidaemia: large-scale observational study. *Thorax* 2016;71(4):347-55. doi: 10.1136/thoraxjnl-2015-207403
25. Zhang C, Yin A, Li H, et al. Dietary Modulation of Gut Microbiota Contributes to Alleviation of Both Genetic and Simple Obesity in Children. *EBioMedicine* 2015;2(8):968-84. doi: 10.1016/j.ebiom.2015.07.007 [published Online First: 2015/10/02]
26. Xu H, Zheng X, Qian Y, et al. Metabolomics Profiling for Obstructive Sleep Apnea and Simple Snorers. *Scientific reports* 2016;6:30958. doi: 10.1038/srep30958 [published Online First: 2016/08/03]
27. Wu SQ, Liao QC, Xu XX, et al. Effect of CPAP therapy on C-reactive protein and cognitive impairment in patients with obstructive sleep apnea hypopnea syndrome. *Sleep & breathing = Schlaf & Atmung* 2016 doi: 10.1007/s11325-016-1331-2 [published Online First: 2016/03/20]
28. Poroyko VA, Carreras A, Khalyfa A, et al. Chronic Sleep Disruption Alters Gut Microbiota, Induces Systemic and Adipose Tissue Inflammation and Insulin Resistance in Mice. *Scientific reports* 2016;6:35405. doi: 10.1038/srep35405 [published Online First: 2016/10/16]

For peer review only

For peer review only



For peer review only - http://bmjopen.bmj.com/site/about/guidelines.xhtml

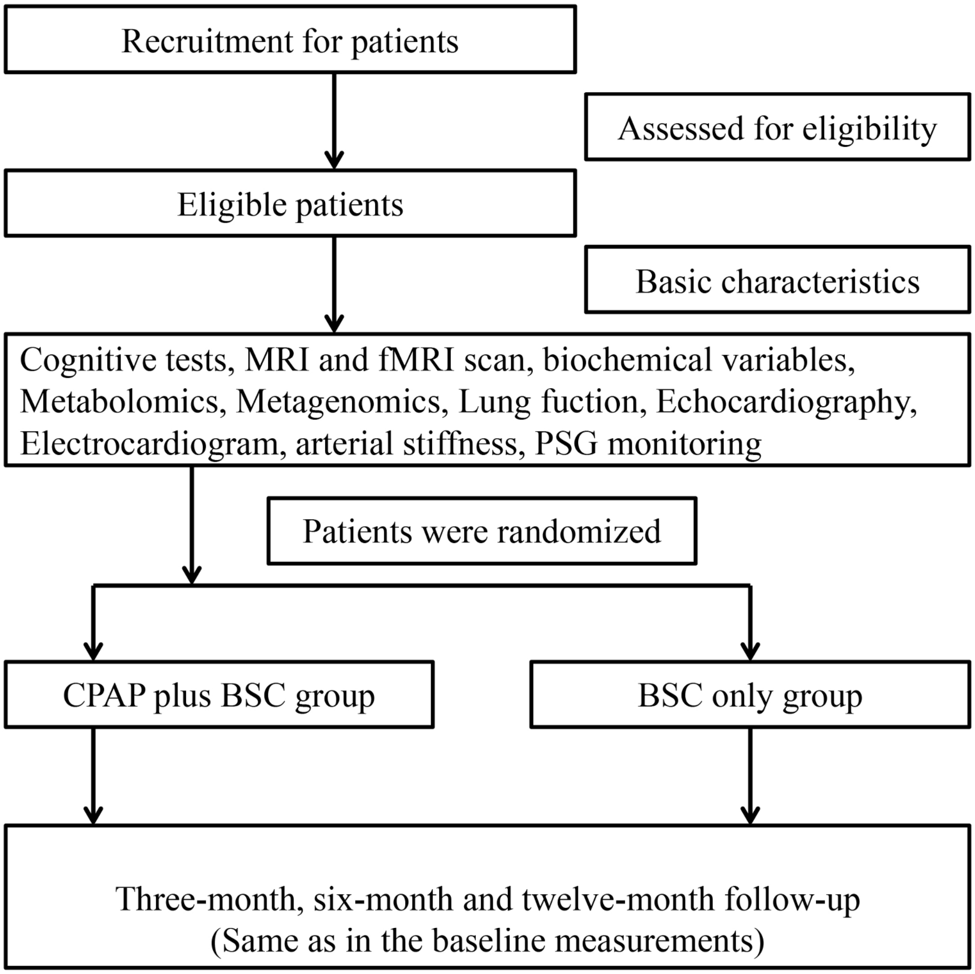


Figure 1. The flow diagram of this RCT
Figure 1
182x190mm (300 x 300 DPI)

# 知情同意书

志愿者姓名： ____________________

亲爱的志愿者，非常感谢您愿意参加我们的多中心临床研究。以下是我们这次研究的一些基本情况。

本次研究由上海交通大学医学院提供研究经费支持（项目号为DLY201502），上海交通大学附属第六人民医院耳鼻咽喉-头颈外科为主要研究中心，上海交通大学附属第一人民医院耳鼻咽喉-头颈外科, 上海交通大学医学院附属仁济医院耳鼻咽喉科, 上海交通大学医学院附属新华医院耳鼻咽喉-头颈外科, 上海交通大学医学院附属第九医院耳鼻咽喉科为研究分中心共同承担本次研究。

一. 研究目的：

此研究是为了更好地了解持续正压通气对阻塞性睡眠呼吸暂停低通气综合征患者中枢神经系统及认知功能的影响

二. 研究周期：

本次研究的时间跨度为1年。在此期间内，我们需要您在我们约定的时间，地点与我们合作。在研究开始前，我们将与您进行约 20 分钟的谈话，以确定您是否适合参加本次研究。

三. 选择条件:（请如实填写您的基本情况研究表并将其交给我们的管理人员）如您有以下状况者，请勿参加本次研究：

1. 未经过标准多导睡眠图检测；

2. 年龄：<30岁或>65岁；

3.严重脏器性疾病（如心，肝，肾功能不全），中枢神经系统疾病及其他睡眠障碍疾病；

4.抑郁症，精神分裂症和其他精神类疾病；

5.头部外伤史，脑肿瘤，癫痫，中风，短暂性脑缺血发作，昏迷等中枢神经系统疾患其他睡眠疾病：发作性睡病，失眠，慢性睡眠剥夺，REM行为障碍和不宁腿综合征，中枢性OSAHS或肥胖低通气综合征；

6.酗酒（每周饮酒超过14标准瓶饮料；男性每天超过四瓶或每周超过七瓶；女性每天超过3瓶。标准瓶：一瓶6.8两的啤酒，一杯2.8两的葡萄酒或0.9两的蒸馏酒）或药



For peer review only

瘾；或正在使用精神类药物，镇静剂，麻醉品等；

7.曾经接受过OSAHS治疗：如CPAP，外科手术，和口腔矫治器等；

8.简明精神状态量表 (MMSE) <24；

9.左利手；

10.MRI禁忌症：如幽闭症或体内有金属植入；

11.过去1年内进行过胃肠道手术，除阑尾炎、疝气手术外；

12.妊娠女性；

13.前8周使用抗生素或使用益生菌等肠道菌群调节剂；

14.过去3个月内因下列疾病接受药物治疗的：胆囊炎、胆结石、消化道溃疡、尿路感染、急性肾盂肾炎、膀胱炎；

15.患有肺结核和艾滋病等传染病；

16. 商业驾驶员或被认为有驾驶相关事故风险的人员；

17. 未能提供我们需要的资料而影响结果判断者；

18. 研究者认为有任何不适合参加试验理由者。

四、提供项目咨询：

在第一次约见之前我们将提供一次咨询：在本次咨询，我们将告知您本次研究的基本情况、志愿者的筛选条件及研究方法等，如果您已经完成前期检测，请将相关检测报告带来，以便我们进行筛选；如果没有相关检测报告，您需要自己承担费用，到医院完成检测包括诊断型多导睡眠检测（PSG）、呼吸机压力滴定和顺应性测试等（推荐在上海市第六人民医院耳鼻咽喉头颈外科及各分中心医院完成）；如果您愿意在我院检测，将与其他所有需要检测人员一样常规入院进行睡眠监测及血液学检测，并需要承担相应检查和检测费用。如果您前期表示愿意参加本次多中心研究，我们将可以根据检测结果，决定是否正式通知您参加本次研究免费检测和筛查，此后涉及本次研究检查及检测均为免费。

五、主要研究内容：

本研究为多中心随机对照研究，参加本研究的患者将被随机分配到CPAP治疗组和最佳支持治疗组，随机分配到CPAP治疗组的患者需每晚（每周不少于5天，每天不少于4小时）佩戴呼吸机进行持续正压通气治疗（研究用呼吸机由上海交通大



For peer review only - http://bmjopen.bmj.com/site/about/guidelines.xhtml

学附属第六医院耳鼻咽喉科免费提供）。随机分配到最佳支持治疗组的患者需按照我们提供的最佳支持治疗方案进行治疗。在参加研究前所有的志愿者需进行问卷填写；体检；睡眠监测；临床行为能力测试；和核磁共振检查等。如果您愿意参加我们的研究，并符合我们筛选的条件，我们将约定下一次见面的时间。

六、约见过程：（我们在约见期间安排的所有检查项目均免费）

（1）第一次约见：

第一次约见：在本次约见中我们将评估您的呼吸机使用情况，进行心超，动脉粥样硬化及临床行为能力测试，核磁共振等检查。

第二次约见（3个月时）：在本次约见中，主要进行随访，具体随访项目包括问卷填写；体检；临床行为能力测试；血液学检测和核磁共振检查等。

第三次约见（6个月时）：在本次约见中，我们主要进行随访，具体随访项目包括问卷填写；体检；临床行为能力测试；血液学检测和核磁共振检查等。

第四次约见（1年时）：在本次约见中，我们主要进行随访，具体随访项目包括。问卷填写；体检；临床行为能力测试；血液学检测和核磁共振检查等。

七、.安全性问题：

呼吸机是目前治疗OSAHS的重要方法和金标准，本次研究涉及的检查及治疗不会对您的身体产生不良影响。

八、.参加研究的风险和收益：

（1）本研究在开始前已经上海市第六人民医院伦理委员会审核通过，无论您被分配到CPAP治疗组，还是最佳支持治疗组，您都将免费获得研究用呼吸机一台，其中被随机分配到单纯最佳支持治疗组的患者，您在研究结束，完成最后一次随访时，根据合同，我们将给予您免费获得研究用呼吸机一台。您和其他志愿者参与本研究，将接受定期医学及健康检查，并从治疗中获益，同时，将有可能为睡眠呼吸暂停低通气综合征的医学诊疗技术发展做出重要的贡献。

（2）无论在任何时候，您都有权利退出本次研究，无须任何解释。根据约定，您退还本研究使用呼吸机，我们将退还押金。但请注意：呼吸机加支持治疗组，因人为原因损坏呼吸机，遗失呼吸机及故意不配合本项研究（如呼吸机外借他人使用，隐瞒既往特殊用药及治疗情况）等严重违反本次实验要求的情况，我们将不能退还



For peer review only - http://bmjopen.bmj.com/site/about/guidelines.xhtml

押金）。

（3）在研究开始前，请如实告知您的既往疾病史，以便我们评估您是否适合参与本次研究。

九、医疗与咨询：在研究过程中，对可能产生某些未知的风险，我们将由经验丰富的专业医疗人员监督并提供相应的健康与医疗咨询。如果您对本研究有任何问题，包括您的权益或不良反应的各方面的疑问，您可以随时与我们联系。

十、保密责任：

1. 在整个研究过程中，我们将对您的身份信息和实验数据信息进行保密。
2. 根据您的意愿，我们将对您认为需保密的其它信息进行保密。
3. 本次研究的总体结果可能会因科学研究的目的而被发表（个人信息将会保密）。

十一、费用说明：为感谢您支持并参加本次多中心研究，我们将为您申请并支付购买本研究使用的莲花牌呼吸机一台，并在本次研究结束后，该呼吸机将免费赠送给您。您正式参加本次多中心研究后，您将在这些健康体检中获益，并且我们安排每次访视的检测项目均免费。

十二、自愿原则：

本次研究按照完全自愿的原则招募志愿者，在研究过程中任何时候您都可以选择退出这次研究，这不会导致您在诊断治疗过程中受到任何歧视与不公平待遇。

项目联系人姓名：许华俊（15901975812）；王慧（18930170357）

2017年 3月12日



For peer review only

For peer review only - http://bmjopen.bmj.com/site/about/guidelines.xhtml

# 志愿者申明

今天我收到了一份关于此次研究的文字说明，它包括：本次研究的目的、过程及对我的要求；参加本次研究安全性方面的后备措施，对治疗的影响及保密措施等内容。

本次研究的负责人或指定人员已经向我说明了该研究的性质，并圆满回答了我的问题。

我知道我并非被迫参加这次研究，而且我可以在任何时候退出这次研究，并不影响我的正常接受诊疗的权利。我知道如果专家认为退出本次研究将更符合我 的利益，那我也可能会被终止参加该研究。

我知道参加本次研究的补偿标准。作为该研究的参加者，我完全理解此项工作，并完全接受上述方案，我承诺将会遵守该研究方案，履行志愿者相关义务。

据我所知，我符合选择标准。我愿意参加该研究。

志愿者签名：________________联系方式：______________ 日期：____________

研究者签名：________________联系方式：______________ 日期：____________



For peer review only - http://bmjopen.bmj.com/site/about/guidelines.xhtml

STANDARD PROTOCOL ITEMS: RECOMMENDATIONS FOR INTERVENTIONAL TRIALS

SPIRIT 2013 Checklist: Recommended items to address in a clinical trial protocol and related documents*

| Section/item | Item No | Description | |
|---|---|---|---|
| **Administrative information** | | | |
| Title | 1 | Descriptive title identifying the study design, population, interventions, and, if applicable, trial acronym | 1 |
| Trial registration | 2a | Trial identifier and registry name. If not yet registered, name of intended registry | 4 |
| | 2b | All items from the World Health Organization Trial Registration Data Set | 4 |
| Protocol version | 3 | Date and version identifier | |
| Funding | 4 | Sources and types of financial, material, and other support | 24 |
| Roles and responsibilities | 5a | Names, affiliations, and roles of protocol contributors | 1-2, 24-25 |
| | 5b | Name and contact information for the trial sponsor | 24 |
| | 5c | Role of study sponsor and funders, if any, in study design; collection, management, analysis, and interpretation of data; writing of the report; and the decision to submit the report for publication, including whether they will have ultimate authority over any of these activities | |
| | 5d | Composition, roles, and responsibilities of the coordinating centre, steering committee, endpoint adjudication committee, data management team, and other individuals or groups overseeing the trial, if applicable (see Item 21a for data monitoring committee) | |
| **Introduction** | | | |
| Background and rationale | 6a | Description of research question and justification for undertaking the trial, including summary of relevant studies (published and unpublished) examining benefits and harms for each intervention | 5 |
| | 6b | Explanation for choice of comparators | |
| Objectives | 7 | Specific objectives or hypotheses | 5,6 |

For peer review only

| | | | |
|---|---|---|---|
| Trial design | 8 | Description of trial design including type of trial (eg, parallel group, crossover, factorial, single group), allocation ratio, and framework (eg, superiority, equivalence, noninferiority, exploratory) | 6 |
| **Methods: Participants, interventions, and outcomes** | | | |
| Study setting | 9 | Description of study settings (eg, community clinic, academic hospital) and list of countries where data will be collected. Reference to where list of study sites can be obtained | 7 |
| Eligibility criteria | 10 | Inclusion and exclusion criteria for participants. If applicable, eligibility criteria for study centres and individuals who will perform the interventions (eg, surgeons, psychotherapists) | 8,9, 10 |
| Interventions | 11a | Interventions for each group with sufficient detail to allow replication, including how and when they will be administered | 11, 12 |
| | 11b | Criteria for discontinuing or modifying allocated interventions for a given trial participant (eg, drug dose change in response to harms, participant request, or improving/worsening disease) | 12 |
| | 11c | Strategies to improve adherence to intervention protocols, and any procedures for monitoring adherence (eg, drug tablet return, laboratory tests) | 12 |
| | 11d | Relevant concomitant care and interventions that are permitted or prohibited during the trial | |
| Outcomes | 12 | Primary, secondary, and other outcomes, including the specific measurement variable (eg, systolic blood pressure), analysis metric (eg, change from baseline, final value, time to event), method of aggregation (eg, median, proportion), and time point for each outcome. Explanation of the clinical relevance of chosen efficacy and harm outcomes is strongly recommended | 13-19 |
| Participant timeline | 13 | Time schedule of enrolment, interventions (including any run-ins and washouts), assessments, and visits for participants. A schematic diagram is highly recommended (see Figure) | 7,8, Figure 1 |
| Sample size | 14 | Estimated number of participants needed to achieve study objectives and how it was determined, including clinical and statistical assumptions supporting any sample size calculations | 20 |
| Recruitment | 15 | Strategies for achieving adequate participant enrolment to reach target sample size | 10 |
| **Methods: Assignment of interventions (for controlled trials)** | | | |
| Allocation: | | | 11 |

| | | | |
|---|---|---|---|
| Sequence generation | 16a | Method of generating the allocation sequence (eg, computer-generated random numbers), and list of any factors for stratification. To reduce predictability of a random sequence, details of any planned restriction (eg, blocking) should be provided in a separate document that is unavailable to those who enrol participants or assign interventions | 11 |
| Allocation concealment mechanism | 16b | Mechanism of implementing the allocation sequence (eg, central telephone; sequentially numbered, opaque, sealed envelopes), describing any steps to conceal the sequence until interventions are assigned | 11 |
| Implementation | 16c | Who will generate the allocation sequence, who will enrol participants, and who will assign participants to interventions | 11 |
| Blinding (masking) | 17a | Who will be blinded after assignment to interventions (eg, trial participants, care providers, outcome assessors, data analysts), and how | 11, 12 |
| | 17b | If blinded, circumstances under which unblinding is permissible, and procedure for revealing a participant's allocated intervention during the trial | |
| **Methods: Data collection, management, and analysis** | | | |
| Data collection methods | 18a | Plans for assessment and collection of outcome, baseline, and other trial data, including any related processes to promote data quality (eg, duplicate measurements, training of assessors) and a description of study instruments (eg, questionnaires, laboratory tests) along with their reliability and validity, if known. Reference to where data collection forms can be found, if not in the protocol | 12-19 |
| | 18b | Plans to promote participant retention and complete follow-up, including list of any outcome data to be collected for participants who discontinue or deviate from intervention protocols | 12 |
| Data management | 19 | Plans for data entry, coding, security, and storage, including any related processes to promote data quality (eg, double data entry; range checks for data values). Reference to where details of data management procedures can be found, if not in the protocol | 21 |
| Statistical methods | 20a | Statistical methods for analysing primary and secondary outcomes. Reference to where other details of the statistical analysis plan can be found, if not in the protocol | 20 |
| | 20b | Methods for any additional analyses (eg, subgroup and adjusted analyses) | 21 |
| | 20c | Definition of analysis population relating to protocol non-adherence (eg, as randomised analysis), and any statistical methods to handle missing data (eg, multiple imputation) | 21 |

**Methods: Monitoring**

| | | | |
|---|---|---|---|
| Data monitoring | 21a | Composition of data monitoring committee (DMC); summary of its role and reporting structure; statement of whether it is independent from the sponsor and competing interests; and reference to where further details about its charter can be found, if not in the protocol. Alternatively, an explanation of why a DMC is not needed | 21 |
| | 21b | Description of any interim analyses and stopping guidelines, including who will have access to these interim results and make the final decision to terminate the trial | 22 |
| Harms | 22 | Plans for collecting, assessing, reporting, and managing solicited and spontaneously reported adverse events and other unintended effects of trial interventions or trial conduct | 22 |
| Auditing | 23 | Frequency and procedures for auditing trial conduct, if any, and whether the process will be independent from investigators and the sponsor | |

**Ethics and dissemination**

| | | | |
|---|---|---|---|
| Research ethics approval | 24 | Plans for seeking research ethics committee/institutional review board (REC/IRB) approval | 21 |
| Protocol amendments | 25 | Plans for communicating important protocol modifications (eg, changes to eligibility criteria, outcomes, analyses) to relevant parties (eg, investigators, REC/IRBs, trial participants, trial registries, journals, regulators) | 25 |
| Consent or assent | 26a | Who will obtain informed consent or assent from potential trial participants or authorised surrogates, and how (see Item 32) | 22 |
| | 26b | Additional consent provisions for collection and use of participant data and biological specimens in ancillary studies, if applicable | 22 |
| Confidentialit y | 27 | How personal information about potential and enrolled participants will be collected, shared, and maintained in order to protect confidentiality before, during, and after the trial | 25 |
| Declaration of interests | 28 | Financial and other competing interests for principal investigators for the overall trial and each study site | 25 |
| Access to data | 29 | Statement of who will have access to the final trial dataset, and disclosure of contractual agreements that limit such access for investigators | 24 |
| Ancillary and post-trial care | 30 | Provisions, if any, for ancillary and post-trial care, and for compensation to those who suffer harm from trial participation | |

For peer review only

| | | | |
|---|---|---|---|
| Dissemination policy | 31a | Plans for investigators and sponsor to communicate trial results to participants, healthcare professionals, the public, and other relevant groups (eg, via publication, reporting in results databases, or other data sharing arrangements), including any publication restrictions | 22 |
| | 31b | Authorship eligibility guidelines and any intended use of professional writers | |
| | 31c | Plans, if any, for granting public access to the full protocol, participant-level dataset, and statistical code | 22 |
| **Appendices** | | | |
| Informed consent materials | 32 | Model consent form and other related documentation given to participants and authorised surrogates | 25 |
| Biological specimens | 33 | Plans for collection, laboratory evaluation, and storage of biological specimens for genetic or molecular analysis in the current trial and for future use in ancillary studies, if applicable | |

*It is strongly recommended that this checklist be read in conjunction with the SPIRIT 2013 Explanation & Elaboration for important clarification on the items. Amendments to the protocol should be tracked and dated. The SPIRIT checklist is copyrighted by the SPIRIT Group under the Creative Commons "Attribution-NonCommercial-NoDerivs 3.0 Unported" license.

For peer review only
